# MicroRNAs and Target Genes As Biomarkers for the Diagnosis of Early Onset of Parkinson Disease

**DOI:** 10.3389/fnmol.2017.00352

**Published:** 2017-10-31

**Authors:** Ahmad R. Arshad, Siti A. Sulaiman, Amalia A. Saperi, Rahman Jamal, Norlinah Mohamed Ibrahim, Nor Azian Abdul Murad

**Affiliations:** ^1^UKM Medical Centre, UKM Medical Molecular Biology Institute, Universiti Kebangsaan Malaysia, Bandar Tun Razak, Malaysia; ^2^Department of Medicine, Faculty of Medicine, UKM Medical Centre, Universiti Kebangsaan Malaysia, Bandar Tun Razak, Malaysia

**Keywords:** Parkinson's disease (PD), microRNA (miRNA), PD related genes, biomarkers, early onset

## Abstract

Among the neurodegenerative disorders, Parkinson's disease (PD) ranks as the second most common disorder with a higher prevalence in individuals aged over 60 years old. Younger individuals may also be affected with PD which is known as early onset PD (EOPD). Despite similarities between the characteristics of EOPD and late onset PD (LODP), EOPD patients experience much longer disease manifestations and poorer quality of life. Although some individuals are more prone to have EOPD due to certain genetic alterations, the molecular mechanisms that differentiate between EOPD and LOPD remains unclear. Recent findings in PD patients revealed that there were differences in the genetic profiles of PD patients compared to healthy controls, as well as between EOPD and LOPD patients. There were variants identified that correlated with the decline of cognitive and motor symptoms as well as non-motor symptoms in PD. There were also specific microRNAs that correlated with PD progression, and since microRNAs have been shown to be involved in the maintenance of neuronal development, mitochondrial dysfunction and oxidative stress, there is a strong possibility that these microRNAs can be potentially used to differentiate between subsets of PD patients. PD is mainly diagnosed at the late stage, when almost majority of the dopaminergic neurons are lost. Therefore, identification of molecular biomarkers for early detection of PD is important. Given that miRNAs are crucial in controlling the gene expression, these regulatory microRNAs and their target genes could be used as biomarkers for early diagnosis of PD. In this article, we discussed the genes involved and their regulatory miRNAs, regarding their roles in PD progression, based on the findings of significantly altered microRNAs in EOPD studies. We also discussed the potential of these miRNAs as molecular biomarkers for early diagnosis.

## Introduction

Parkinson's disease (PD) is the second most common neurodegenerative disorder with an approximate incidence of 1:800–1,000 in subjects aged over 60 years old (Bekris et al., 2010; Lin and Farrer, 2014). PD is a progressive disorder that affects movement that is often diagnosed based on the presence of motor signs such as tremor, bradykinesia, muscle rigidity, and postural instability though in some PD patients, non-motor symptoms of anxiety, fatigue, depression, sleep disturbance, gastrointestinal, and sexual dysfunctions are also observed (Schneider and Obeso, 2015). As PD is an age-related disorder, it is more common in the elderly; however younger individuals may also be affected (Gasser et al., 2011) and these patients are usually known as early onset PD (EOPD) patients. The typical PD, or also known as late onset PD (LOPD), is defined for individuals more than 50 years old who exhibit PD signs and symptoms (Schrag and Schott, 2006). Some of EOPD patients show slower disease progression and worse disease outcomes in comparison to LOPD patients (Schrag et al., 1998; Inzelberg et al., 2004; Ferguson et al., 2015), implying that there could be biological differences that dictates the disease outcomes and also the age of onset of PD. There are some evidence that EOPD may be associated with some genetic alterations in the PD progression (Oki et al., 2016), yet the molecular mechanisms to differentiate the EOPD and LOPD progression remains unclear and requires further understanding.

Parkinson's disease (PD) is a multi-factorial disease involving interactions between environment and genetic factors (Farrer, 2006; Sellbach et al., 2006). Initially, PD is thought to be a sporadic disease due to environmental factors such as exposure to toxins namely, paraquat, 1-methyl-4-phenyl-1,2,3,6-tetrahyropyridine (MPTP) or pesticides (Langston et al., 1983; Ascherio and Schwarzschild, 2016). However, in the past few decades, genetic inheritance has been also identified to be one of the causative factors that could contribute to PD progression (Gasser et al., 2011; Lin and Farrer, 2014). About 90% of PD cases are sporadic whereas >10% of PD patients have a family history with the contribution of one or several genetic alterations (Klein and Westenberger, 2012; Ascherio and Schwarzschild, 2016). These genetic alterations in PD can be categorized into autosomal dominant (AD) and autosomal recessive (AR) of PD (Bekris et al., 2010; Klein and Westenberger, 2012). The AD form of PD is associated with alterations of the α-synuclein (*SNCA*) and leucine rich repeat kinase 2 (*LRRK2*) genes (Bekris et al., 2010; Klein and Westenberger, 2012) whereas the Parkin RBR E3 ubiquitin protein ligase (*PRKN)*, Parkinsonism associated deglycase (*PARK7*), PTEN induced putative kinase 1 (*PINK1*) and ATPase 13A2 (*ATP13A2*) genes are linked to the AR form of PD (Nuytemans et al., 2010; Heman-Ackah et al., 2013). Current effective treatments for both motor and non-motor symptoms in PD are available, yet the heterogeneous nature of this disease causes diverse clinical manifestations across the PD patients (Oertel and Schulz, 2016) reducing the effectiveness of those treatments. Moreover, the underlying mechanisms of PD are still poorly understood and the lack of effective approach to differentiate and identify the EOPD and LOPD patients also contribute to the complexity of PD management. Therefore, finding the early diagnostic approaches for EOPD and LOPD are essential for better disease management.

Biological markers, or biomarkers like proteins or other molecular elements which are expressed by cells and tissues can reflect the pathological processes underlying PD progression. The fact that these biomarkers can readily be found in body fluids, such as in blood, urine, or even in cerebrospinal fluid (CSF) (Gwinn et al., 2017) therefore they are the most suitable biomarkers which can accurately diagnose the disease and symptoms, as well as predicting the severity and progression. One such biomarker is microRNAs (miRNAs), in which the recent discovery of miRNAs involvement in PD has garnered interest especially their roles in disease progression (Serafin et al., 2014). miRNAs are single non-coding small RNAs (19–24 nucleotides), which are able to regulate gene expression by mRNA repression or mRNA cleavage (Bartel, 2004). Compared to other organs, there are more miRNAs expressed in the human brain, in which previous studies observed that the function of these brain miRNAs are not only restricted to cell fate determination (Meza-Sosa et al., 2014), but are also involved in neuroplasticity and neurobiological functions (Qiu et al., 2015; Batistela et al., 2017). Dysregulation of these miRNAs leads to mitochondrial dysfunction, altered mitochondrial dynamics, accumulation of fragmented mitochondria, oxidative stress, excitotoxicity, cell death and α-synuclein aggregation, thus subsequently causing neurodegeneration (Martin, 2010; Spano et al., 2015). Since a single miRNA can regulate different genes and a single target gene may also be regulated by multiple miRNAs (Qiu et al., 2015), this network regulation of miRNAs and their complementary target mRNAs may provide additional insights on the disease progression and possible new therapeutic approaches. Given these interests, this mini review aimed to discuss the role of PD related genes and their regulatory microRNAs to differentiate the early-onset (EOPD) and late-onset PD (LOPD). The applications and challenges of using these microRNAs as potential biomarkers are also discussed.

## Early and late onset of parkinson disease

Within the PD patients, those who are below 50 years old when they first exhibited the PD symptoms are classified as EOPD, which is less common compared to LOPD, or typical PD (Schrag et al., 1998; Schrag and Schott, 2006). The mortality of EOPD is two times greater than typical PD cases (Schrag et al., 1998). EOPD patients experience a longer disease course, with slower disease progression and cognitive decline, yet some motor complications such as dyskinesia and dystonia have been observed to develop earlier than LOPD (Schrag et al., 1998; Inzelberg et al., 2004; Ferguson et al., 2015), consequently causing a significant impairment in the quality of life. In fact, a previous study showed that between EOPD and LOPD patients, the dopaminergic neurons (DA) loss is greater in the EOPD group (Fereshtehnejad et al., 2014), implying that there are differences in the underlying pathophysiology and molecular mechanisms. However, more definitive and confirmatory studies are needed to differentiate between these two subsets of PD patients for better management and personalized treatment.

Parkinson's disease (PD) symptoms are usually present when about 70–80% of the dopaminergic (DA) neurons are lost (Goldenberg, 1990). The neuropathological hallmark of PD is the deposition of the intracytoplasmic protein inclusions known as Lewy bodies (LB) that are composed of various molecules including α-synuclein (protein product of *SNCA* gene) aggregates, which is responsible for the loss of DA neurons in the midbrain of substantia nigra (Spillantini et al., 1997; Wakabayashi et al., 2007; Longhena et al., 2017). Currently, there is no test or examination that can be performed to diagnose PD at an early stage accurately. The standard approach for PD diagnosis is based on the patient's and family history, and on neurological examination to demonstrate the presence of the common signs and symptoms including bradykinesia, rigidity, and tremor (Massano and Bhatia, 2012). Magnetic resonance imaging has also been used as a tool to analyse for brain abnormalities in which multisystem atrophy, progressive supranuclear palsy or cortical degeneration were identified to differentiate between PD and atypical parkinsonism (Ba and Martin, 2015). However, the sensitivity for this MRI diagnosis was only around 60–80% (Kraft et al., 2002; Feng et al., 2015; Hwang et al., 2015) thus limiting its diagnostic value. Nevertheless, the confirmative diagnosis of PD can only be done through a neuropathological investigation by detecting the loss of DA neurons and the presence of LB and Lewy neurites in the brain (Braak et al., 2003; Massano and Bhatia, 2012).

There is currently no single genetic test to accurately identify those who are susceptible to PD. However, recent findings showed that some genetic markers can be used to diagnose EOPD. Genes such as *PRKN, PARK7, PINK1, LRRK2*, and Glucosylceramidase beta (*GBA*) have been consistently shown to be associated with age of onset in PD exclusively (Klein and Westenberger, 2012; Lin and Farrer, 2014). Several genetic mutations in *PRKN* have been associated with EOPD (Deng et al., 2008; Chen H. et al., 2016) in which the frequency of this gene mutations in the EOPD population is about 7.2–12.5% (Hardy et al., 2009). In fact, mutations in *PRKN* gene are the most common cause for EOPD that account for 50% of those EOPD patients (onset age <25 years) and about 3–7% for EOPD patients (onset age = 30–45 years) (Lücking et al., 2000; Periquet et al., 2003). Thus, *PRKN* gene is likely to be the main player of genetic susceptibility in PD progression at a much earlier age. A more rare genetic association was observed for *PINK1* gene, in which the mutations in *PINK1* gene is responsible for about 2–4% of EOPD cases in Caucasians (Valente et al., 2004; Bonifati et al., 2005) and 4–9% in Asian populations (Li et al., 2005; Tan et al., 2006). Similarly, the mutations in *PARK7* are also rare in EOPD that represent about 1% of the total EOPD cases (Abou-Sleiman et al., 2003; Hague et al., 2003), yet the inheritance of these homozygous or heterozygous mutations of *PARK7* are almost 100% (Schulte and Gasser, 2011), therefore making *PARK7* gene as equally important as *PRKN* gene. On the other hand, *GBA* and *LRKK2* mutations are generally associated with typical PD with the late-onset symptoms, though severe mutations in *GBA* gene tended to be associated with much early age of the PD onset (Lesage et al., 2011). About 8% of the *LRRK2* mutation carriers were EOPD patients (Healy et al., 2008). Therefore, these genes may offer potential diagnostic values in predicting the EOPD and differentiate them from the typical late-onset PD. Nevertheless, these genetic mutations alone (DNA biomarker) may not be enough to differentiate EOPD from LOPD due to PD heterogeneity and their prevalence in the populations. Thus, a combination of these mutated genes as well other related genes and their respective regulatory microRNAs (stable RNA biomarker) should offer a better diagnostic option as they reflect the actual dysregulation of the cellular states and disease progression of EOPD; hence allowing for a sensitive genetic testing to differentiate EOPD from LOPD.

## microRNA regulatory network in parkinson disease

**Figure 1:**
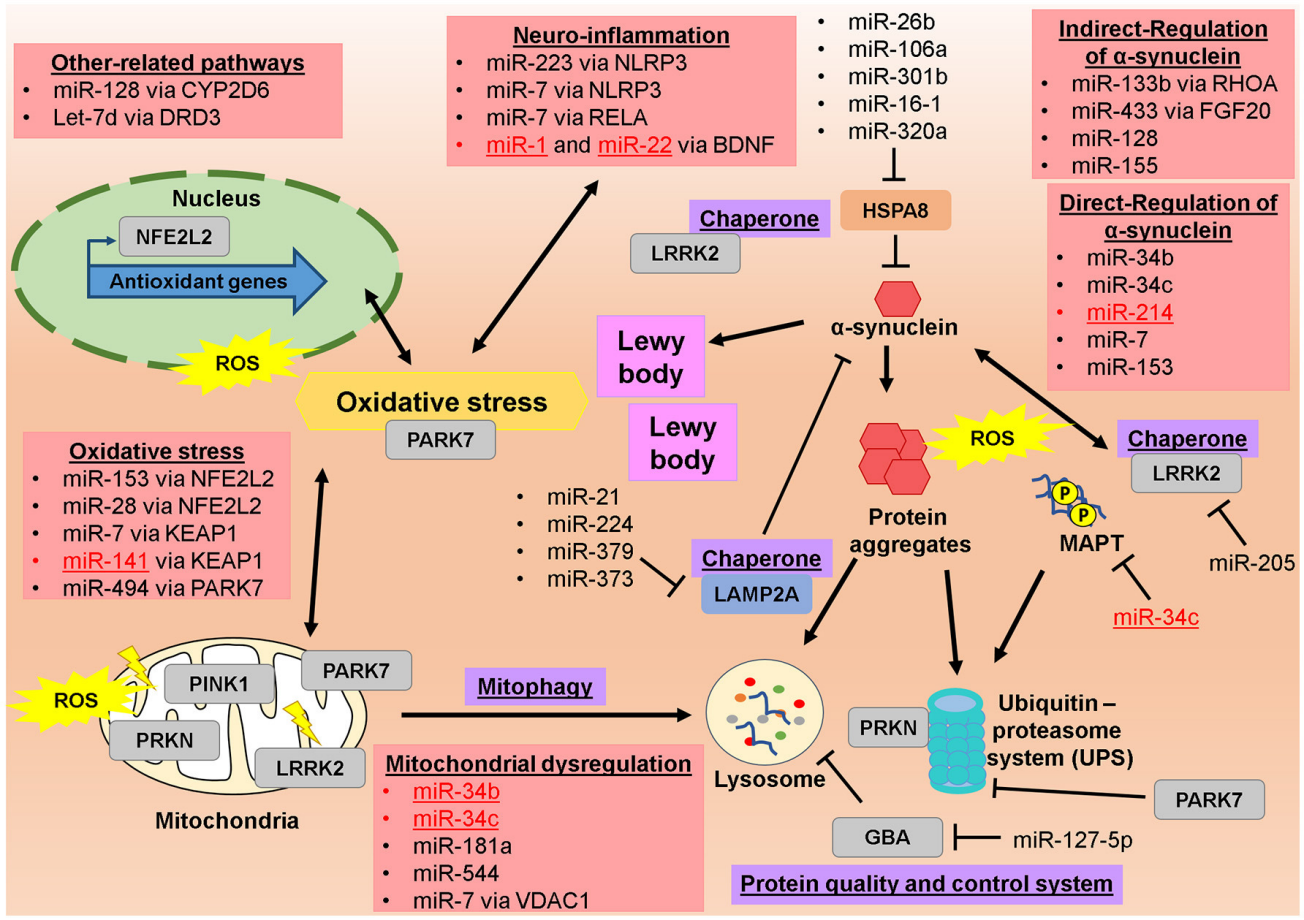
Illustrated regulatory network of altered microRNAs and their targeted genes in pathophysiology of Parkinson's disease. Altered microRNAs from EOPD and LOPD studies are significantly involved in the regulating various molecules in pathophysiological of PD, particularly in mitochondrial dysfunction, oxidative stress, neuro-inflammation, and toxic protein accumulation. Red-colored microRNAs are significantly altered in EOPD patients. Genes full names are in the appendix.

The microRNA regulatory network in PD has been discussed before, particularly in the potential of miRNAs as circulating biomarkers for the diagnosis and treatment of PD (dos Santos et al., 2016; Ma et al., 2016; Marques et al., 2016; Mushtaq et al., 2016; Batistela et al., 2017), in PD progression (Heman-Ackah et al., 2013) as well as in the oxidative stress pathway (Qiu et al., 2014; Xie and Chen, 2016). However, none of these discussions emphasis on the potential of these miRNAs to differentiate the EOPD from LOPD. From 2011 till now, there were few studies that investigated the miRNAs profile in the EOPD patients and compared them to LOPD patients in which lots of LOPD studies did not include the EOPD comparison (Table 1). Among these identified miRNAs, few of them showed a promise as a potential biomarker for EOPD especially, when their validated targets/genes are involved in PD progression (Figure 1). Among them, miR-34b and miR-34c have been shown to be decreased in the brain tissues in both EOPD and LOPD patients (Miñones-Moyano et al., 2011). Since miR-34b and miR-34c are shown to target multiple genes in PD including *PRKN* and *PARK7* (Miñones-Moyano et al., 2011), *SNCA* (Kabaria et al., 2015b), *MAPT* (Wu H. et al., 2013), these miRNAs are potentially useful to detect early dysregulation and onset of PD progression. Importantly, miR-331-5p is the only miRNAs that has been identified in plasma of EOPD patients exclusively, with its expression was increased in EOPD patients with no difference was observed in LOPD (Cardo et al., 2013). However, no validated targets/genes were identified for miR-331-5p particularly in PD progression, thus these would require further investigation. From these findings, they implied that the role of miRNAs in PD disease progression is significant and thus could also play a role in the determining the age-onset of PD symptoms. Therefore, the potential roles of these miRNAs and their target genes in differentiating EOPD from LOPD will be discussed below, particularly by looking at the various PD pathological events.

**Table 1:** Summary of the altered microRNAs and their targeted genes in Parkinson Disease (PD) with a focus on EOPD and LOPD patients.

**Diagnosis**	**Sources**	**miRNAs**	**miRNA expression in EOPD**	**miRNA expression in LOPD**	**Validated target genes associated with PD**
EOPD	Whole Blood (Margis et al., 2011)	miR-1	Reduced	NS	*BDNF* (Brandenburger et al., 2014; Varendi et al., 2014)
		miR-22	Reduced	NS	*BDNF* (Muiños-Gimeno et al., 2011)
		miR-29a	Reduced	Reduced	–
	Brain Tissues (Miñones-Moyano et al., 2011)	miR-34b,c	Reduced	Reduced	*PRKN* and *PARK7* (Miñones-Moyano et al., 2011), *SNCA* (Kabaria et al., 2015b), *MAPT* (Wu H. et al., 2013)
	Plasma (Cardo et al., 2013)	miR-331-5p	Increased	NS	–
	Serum (Dong et al., 2016)	miR-141	Reduced	Reduced	*KEAP1* (Shi et al., 2015; Wang et al., 2016; Cheng et al., 2017)
		miR-146b-5p	Reduced	Reduced	–
		miR-193a-3p	Reduced	Reduced	–
		miR-214	Reduced	Reduced	*SNCA* (Wang Z. H. et al., 2015)
LOPD	Whole Blood (Serafin et al., 2015)	miR-103a	NC	Increased	–
		miR-29a	NC	Increased	–
		miR-30b	NC	Increased	–
	Whole Blood (Yilmaz et al., 2016)	miR-3143	NC	Reduced	–
		miR-335-3p	NC	Reduced	–
		miR-4671-3p	NC	Reduced	–
		miR-561-3p	NC	Reduced	–
		miR-579-3p	NC	Reduced	–
	Brain tissues	miR-34b,c	Reduced	Reduced	*PRKN* and *PARK7* (Miñones-Moyano et al., 2011), *SNCA* (Kabaria et al., 2015b), *MAPT* (Wu H. et al., 2013)
	Brain tissue (Liao et al., 2013)	miR-181a,b,c,d	NC	Reduced	*PRKN* (Cheng et al., 2016)
		miR-22	NC	Reduced	*BDNF* (Muiños-Gimeno et al., 2011)
		miR-29a,b,c	NC	Reduced	–
	Brain tissue (Alvarez-Erviti et al., 2013)	miR-106a	NC	Increased	*HSPA8* (Alvarez-Erviti et al., 2013)
		miR-21	NC	Increased	*LAMP2A* (Alvarez-Erviti et al., 2013; Su et al., 2016), *PPARA* (Fu et al., 2017)
		miR-224	NC	Increased	*LAMP2A* (Alvarez-Erviti et al., 2013), NPAS4 (Choy et al., 2017)
		miR-26b	NC	Increased	*HSPA8* (Alvarez-Erviti et al., 2013), *RB1* (Absalon et al., 2013), *BDNF* (Caputo et al., 2011)
		miR-301b	NC	Increased	*HSPA8* (Alvarez-Erviti et al., 2013)
		miR-373	NC	Increased	*LAMP2A* (Alvarez-Erviti et al., 2013)
	Brain tissue (Cho et al., 2013)	miR-205	NC	Reduced	*LRRK2* (Cho et al., 2013)
	Brain tissue (Cardo et al., 2014)	miR-135b	NC	Reduced	–
		miR-198	NC	Reduced	–
		miR-485-5p	NC	Reduced	–
		miR-548d	NC	Increased	–
	Brain tissue (Hoss et al., 2016)	Let-7i-3p/5p	NC	Reduced	–
		miR-10b-5p	NC	Reduced	BDNF (Varendi et al., 2014)
		miR-1224	NC	Reduced	–
		miR-127-3p	NC	Reduced	–
		miR-127-5p	NC	Increased	*GBA* (Siebert et al., 2014)
		miR-16-5p	NC	Increased	–
		miR-184	NC	Reduced	–
		miR-29a-3p	NC	Increased	–
	Brain tissues (Tatura et al., 2016)	miR-144	NC	Increased	–
		miR-145	NC	Reduced	–
		miR-199b	NC	Increased	–
		miR-221	NC	Increased	–
		miR-488	NC	Increased	–
		miR-543	NC	Reduced	–
		miR-544	NC	Increased	*PARK7* (Jin et al., 2016)
		miR-7	NC	Reduced	*SNCA* (Junn et al., 2009; Doxakis, 2010),*NLRP3* (Zhou et al., 2016), *RELA* (Choi et al., 2014), *KEAP1* (Kabaria et al., 2015a), *VDAC1* (Chaudhuri et al., 2016)
	Brain tissues (Wake et al., 2016)	miR-225	NC	Reduced	–
		miR-236	NC	Increased	–
		miR-46	NC	Increased	–
	CSF (Burgos et al., 2014)	Let-7g-3p	NC	Increased	–
		miR-1224-5p	NC	Reduced	–
		miR-127-3p	NC	Reduced	–
		miR-128	NC	Reduced	*CYP2D6* (Li et al., 2015)
		miR-132-5p	NC	Reduced	–
		miR-19a,b	NC	Increased	–
		miR-212-3p	NC	Reduced	–
		miR-370	NC	Reduced	–
		miR-409-3p	NC	Reduced	–
		miR-4448	NC	Reduced	–
		miR-485-5p	NC	Reduced	–
		miR-873-3p	NC	Reduced	–
	CSF (Gui et al., 2015)	Let-7g-3p	NC	Increased	–
		miR-1	NC	Reduced	*BDNF* (Brandenburger et al., 2014; Varendi et al., 2014)
		miR-103a	NC	Increased	–
		miR-10a-5p	NC	Increased	–
		miR-119a	NC	Reduced	–
		miR-126	NC	Reduced	–
		miR-127-3p	NC	Increased	–
		miR-132-5p	NC	Increased	–
		miR-136-3p	NC	Increased	–
		miR-151	NC	Reduced	–
		miR-153	NC	Increased	*SNCA* (Doxakis, 2010; Kim et al., 2013; Lim and Song, 2014), *NFE2L2* (Narasimhan et al., 2014; Yang W. et al., 2015)
		miR-16-2	NC	Increased	–
		miR-19b-3p	NC	Reduced	–
		miR-22	NC	Reduced	*BDNF* (Muiños-Gimeno et al., 2011)
		miR-26a	NC	Increased	*BDNF* (Caputo et al., 2011)
		miR-28	NC	Reduced	*NFE2L2* (Yang et al., 2011)
		miR-29a,c	NC	Reduced	–
		miR-301a	NC	Reduced	–
		miR-30b	NC	Increased	–
		miR-331-5p	NC	Increased	–
		miR-370	NC	Increased	–
		miR-374	NC	Reduced	–
		miR-409-3p	NC	Increased	–
		miR-433	NC	Increased	*FGF20* (Wang et al., 2008)
		miR-485-5p	NC	Increased	–
		miR-873-3p	NC	Increased	–
	CSF (Soreq et al., 2013)	miR-1249	NC	Increased	–
		miR-1274b	NC	Increased	–
		miR-150	NC	Increased	–
		miR-16	NC	Reduced	*HSPA8* (Zhang and Cheng, 2014)
		miR-18b	NC	Increased	–
		miR-199b	NC	Increased	–
		miR-20a	NC	Increased	–
		miR-21	NC	Increased	*LAMP2A* (Alvarez-Erviti et al., 2013; Su et al., 2016)
		miR-320a,b	NC	Reduced	*HSPA8* (Li G. et al., 2014)
		miR-378c	NC	Increased	–
		miR-4293	NC	Increased	–
		miR-671	NC	Increased	–
		miR-769	NC	Reduced	–
		miR-92b	NC	Reduced	–
	Plasma (Khoo et al., 2012)	miR-222	NC	Reduced	–
		miR-505	NC	Reduced	–
		miR-626	NC	Reduced	–
	Serum (Botta-Orfila et al., 2014)	miR-19b	NC	Reduced	–
		miR-29a,c	NC	Reduced	–
	Serum (Zhao et al., 2014)	miR-133b	NC	Reduced	*RHOA* (Lu X. C. et al., 2015; Niu et al., 2016)
	Serum (Bai et al., 2017)	miR-29a,b,c	NC	Reduced	–
	Serum (Ma et al., 2016)	miR-146a	NC	Reduced	–
		miR-214	NC	Reduced	*SNCA* (Wang Z. H. et al., 2015)
		miR-221	NC	Reduced	–
		miR-29c	NC	Reduced	–
	Serum (Burgos et al., 2014)	miR-1294	NC	Reduced	–
		miR-16-2-3p	NC	Reduced	–
		miR-30a,e	NC	Increased	*BDNF* (Mellios et al., 2008)
		miR-338-3p	NC	Increased	–
	Serum (Vallelunga et al., 2014)	miR-148b	NC	Reduced	–
		miR-223	NC	Increased	*NLRP3* (Yang Z. et al., 2015)
		miR-24	NC	Increased	–
		miR-30c	NC	Reduced	–
		miR-324-3p	NC	Increased	–
	Serum (Ding et al., 2016)	miR-15b	NC	Reduced	–
		miR-181a	NC	Reduced	*PRKN* (Cheng et al., 2016)
		miR-185	NC	Reduced	–
		miR-195	NC	Increased	*BDNF* (Mellios et al., 2008)
		miR-221	NC	Reduced	–
	PBMCs (Martins et al., 2011)	miR-126	NC	Reduced	–
		miR-126^*^	NC	Reduced	-
		miR-147	NC	Reduced	–
		miR-151-3p,5p	NC	Reduced	–
		miR-199a-3p,5p	NC	Reduced	–
		miR-199b	NC	Reduced	–
		miR-19b	NC	Reduced	–
		miR-26a	NC	Reduced	*BDNF* (Caputo et al., 2011)
		miR-28-5p	NC	Reduced	–
		miR-29b,c	NC	Reduced	–
		miR-301a	NC	Reduced	–
		miR-30b,c	NC	Reduced	–
		miR-335	NC	Reduced	–
		miR-374a,b	NC	Reduced	–

*Summary of the significantly altered microRNAs expressions in EOPD and LOPD studies. All the validated targets/genes for these microRNAs are listed below only in a relation with PD progression or associated pathways where it is possible. EOPD, early-onset PD, LOPD, late-onset PD, NS, Not significant, NC, No comparison was made. Genes full names are in the appendix*.

### MicroRNAs in mitochondrial dysregulation

One of the earliest events in PD pathophysiological processes is the mitochondrial dysregulation (Figure 1). Impairment in oxidative phosphorylation can result in dysregulation of the mitochondrial activity and energy metabolism thus can increase reactive-oxygen-species (ROS) production and oxidative stress, eventually lead to neurotoxicity and death (Dias et al., 2013; Hu and Wang, 2016). This is evident by the reduced of mitochondrial complex I activity observed in PD patients (Schapira et al., 1989) and peroxisome proliferator-activated receptor gamma coactivator 1-alpha (PGC1α), which is an important activator for mitochondrial genes, was decreased in PD patients (Zheng et al., 2010). In fact, experimental studies of using neurotoxin, MPTP (Kolata, 1983) and pesticides like rotenone (Perier et al., 2003) showed similar mitochondrial dysregulation thus, re-affirmed that damaged mitochondria as a key mechanism for DA neuronal impairment. Several keys regulators have been identified in mitochondrial dysregulation in PD, particularly the PRKN and PINK1 interaction (Deas et al., 2011). Previous studies have shown that PINK1 is responsible to detect mitochondrial damage and recruits PRKN enzyme to ubiquitinate the damaged mitochondria (Matsuda et al., 2010; Narendra et al., 2010; Vives-Bauza et al., 2010). This ubiquitination tagging will allow the mitophagy to occur and remove the damaged mitochondria (Deas et al., 2011) from the cytoplasm, therefore preventing the parkinsonism to develop. Moreover, PRKN regulates the Zinc finger protein 746 (also known as PARIS) (Shin et al., 2011), which is a transcriptional repressor of *PGC1*α gene (Scarpulla, 2008). Dyregulation of *PGC1*α expression will affects energy metabolism and mitochondrial biogenesis (Wu et al., 1999). In the progression of PD due to nitrosative stress and ROS, suppression of *PRKN* gene expression resulted in an accumulation of ZNF746 in the cytosol (LaVoie et al., 2005). The formation of the ZNF746 complex selectively downregulates nuclear respiratory factor-1 that controls transcription of the mitochondrial biogenesis, consequently causes DA degeneration (Shin et al., 2011). Besides PRKN and PINK1, PARK7 or also known as protein deglycase (*DJ1*) can bind to mitochondrial complex I subunit, NDUFA4 and NADH dehydrogenase 1 and subsequently prevented PD progression via maintaining the complex 1 activity (Hayashi et al., 2009). Therefore, changes in these genes (*PRKN, PINK1*, and *PARK7*) expression and functions may therefore be indicators of mitochondrial damage and dysregulation that mark the initial progression of PD and neurodegeneration in the patients.

Several studies have implied that miRNAs play crucial role as biomarkers and a therapeutic agent to prevent mitochondrial dysfunction in PD (Figure 1). One such is miR-34b and miR-34c that co-regulate both PARK7 and PRKN, as evidently by the suppression of both miRNAs resulted in a significant reduction in the expression of *PARK7* and *PRKN* (Miñones-Moyano et al., 2011). Previous study involving PD patients at different stages of the disease, showed that there was a downregulation of 40–65% expression of miR-34b and miR-34c in the brain, both in EOPD and LOPD patients (Miñones-Moyano et al., 2011; Table 1). The findings implied that both of these miRNAs may play a role in pathogenesis of PD rather than a consequence of PD progression, since those EOPD patients did not received any drug treatment yet (Miñones-Moyano et al., 2011). In addition, suppression of miR-34b/c expressions in the differentiated SH-SY5Y cells also resulted in a moderate reduction of the cell viability, with the presence of altered mitochondrial function, oxidative stress and also the reduction of the total cellular ATP content in the cells (Ma et al., 2013), confirming that the suppression of miR-34b/c is an indicator of early progression of PD due to mitochondrial damages in the patients, consistent with the reduced expressions of miR-34b/c in both EOPD and LOPD studies (Table 1). Other miRNAs is miR-181a that is highly expressed in the brain (Miska et al., 2004) and can directly regulate *PRKN* gene expression (Cheng et al., 2016). miR-181a expression found to be reduced in the brain (Liao et al., 2013) and serum (Ding et al., 2016) of LOPD patients. Overexpression of miR-181a suppressed *PRKN* expression and partially inhibited the removal of dysfunctional mitochondria by mitophagy (Cheng et al., 2016), thus subsequently leads to disruption in energy maintenance and mitochondrial quality control. Importantly, introduction of PRKN back into the cells eliminated those negative effects of miR-181a (Cheng et al., 2016), implying that an increase of miR-181a expression may reflect the onset of mitochondrial dysregulation consistent with the suppression of PRKN. However, in PD patients, none of the EOPD studies showed changes in miR-181a expression whereas in LOPD patients, miR-181a expression was reduced (Table 1). Another miRNAs is miR-544 that was increased in the brain tissues of LOPD patients (Tatura et al., 2016) and can directly bind to 3′UTR of PARK7 mRNA region to suppress *PARK7* expression (Jin et al., 2016), however, whether miR-544 is implicated in PD progression remains unknown. Another miRNA is miR-7 that regulates *SNCA* expression and α-synuclein level (Junn et al., 2009; Doxakis, 2013). In experimental model of PD, α-synuclein can inhibit mitochondrial complex I activity and causes mitochondrial damage (Martin et al., 2006). This is also showed by an overexpression of pathogenic α-synuclein, A53T/A30P that caused mitochondrial fragmentation via dynamin-like 120 kDa protein, thus implies that α-synuclein also play a role in mitochondrial dysregulation. Moreover, miR-7 also regulates mitochondrial permeability via voltage-dependent anion channel 1 (VDAC1) (Chaudhuri et al., 2016), though how this gene is involved in PD progression particularly in mitochondrial dysregulation would require further studies. Intriguingly, a study of the LRRK2 mice model has identified three differentially expressed miRNAs (miR-16, miR-15a, and miR-21) in *LRRK2*-knockout mice compared to wildtype mice (Dorval et al., 2014). These miRNAs were predicted to affect mitochondrial dysfunction and apoptosis following dysregulation of miR-16/15 expression (Cimmino et al., 2005; Nishi et al., 2010). Interestingly, in this study (Dorval et al., 2014), miR-103 was found to be down-regulated in transgenic mice expressing PD-associated *LRRK2* R1441G mutation when compared to wildtype mice, yet no information of the direct regulation or predicted site of miR-103 in *LRRK2* gene was observed. This miR-103 dysregulation may suggest that there are underlying mechanisms of miR-103 regulation of *LRRK2* gene which may be implicated in PD progression. Previous study showed that elevated level of LRRK2 protein can induce mitochondrial fragmentation via dynamin-like protein (Wang et al., 2012). Nevertheless, some of these miRNAs were altered in the EOPD patients thus may offer diagnostic potential to differentiate EOPD and LOPD, though more information are needed as the comparison for EOPD patients are lacking in those previous studies (Table 1).

### MicroRNAs in α-synuclein regulation and protein control system

SNCA gene encodes the α-synuclein protein and among the important genes that are associated with PD in general, *SNCA* gene is well-recognized as the main player in PD pathogenesis, in which mutations or rearrangements of *SNCA* gene are found in those with autosomal dominant PD (Thomas et al., 2011; Klein and Westenberger, 2012). Genome-wide association studies have demonstrated that *SNCA* gene is significantly associated with PD in multiple populations (Simón-Sánchez et al., 2009; Hamza and Payami, 2010; Tan et al., 2014; Guo et al., 2015; Foo et al., 2017). There is evidence of the *SNCA* gene susceptibility alleles affecting the expression and plasma level of *SNCA* (Bekris et al., 2010; Mata et al., 2010), thus reaffirming the important role of SNCA in PD susceptibility. Structurally, α-synuclein is a protein with a natively unfolded monomer which forms β-sheet-rich soluble oligomers (Outeiro et al., 2009; Stefanis, 2012). Mutated α-synuclein protein has a tendency to aggregate and forms a mature fibril which in turn increases the inclination to form LB (Dawson et al., 2010; Stefanis, 2012). Moreover, the accumulation of transmissible α-synuclein aggregates in a cell can be transferable to another cell thus causing a cumulative risk of the LB spreading across the neurons, and eventually inducing major cells deaths (Dauer and Przedborski, 2003; Recasens et al., 2014). Intriguingly, higher levels of normal non-mutated α-synuclein protein also increases α-synuclein protein aggregation and LB formation in PD patients (Singleton et al., 2003), suggesting that slight changes in *SNCA* gene expression or α-synuclein protein level will trigger PD progression regardless of the mutational status of *SNCA* gene or α-synuclein protein. Therefore, it is important that *SNCA* gene expression is tightly regulated in the brain and hence, miRNA regulatory network of *SNCA* gene expression may play important roles in triggering the PD progression and can be the potential biomarkers to identify EOPD.

Several microRNAs are shown to be able to regulate *SNCA* gene expression (Figure 1; Recasens et al., 2016), and few of them are significantly been altered in EOPD patients (Table 1, Figure 1). Previous study of miRNA expression profiling in brain tissues of PD patients revealed that decreased expression of miR-34b and miR-34c in amygdala region of the brain can be detected in EOPD patients even without any pre-motor symptoms, though similar miR-34b/c reduction was also seen in LOPD patients (Miñones-Moyano et al., 2011). miR-34b/c have been shown to regulate *SNCA* gene expression and α-synuclein protein as well as neuronal toxicity, due to miR-34b, and miR-34c can directly bind to 3′UTR of *SNCA* mRNA (Kabaria et al., 2015b). Inhibition of miR-34b and miR-34c expression in SH-SY5Y neuronal cells caused an increase of α-synuclein protein levels and promoted α-synuclein aggregation (Kabaria et al., 2015b). Importantly, in this study (Kabaria et al., 2015b), a single-nucleotide polymorphism (SNP) in *SNCA* gene, rs10024743 which causes a change of U to G nucleotide at the 3′UTR of *SNCA* mRNA, inhibited the miR-34b binding to *SNCA* mRNA. These findings suggest that the role of genetic susceptibility in *SNCA* gene can alter the outcome of miRNA regulation and subsequently contribute to PD. Besides that, a previous study that used four miRNAs as serum biomarkers to identify EOPD have found that miR-214 was reduced in both EOPD and LOPD patients, when compared to normal controls, yet its expression did not differ between EOPD and LOPD groups (Table 1; Dong et al., 2016). Like miR-34b/c, miR-214 has also can regulate *SNCA* gene expression and α-synuclein protein (Wang Z. H. et al., 2015). In fact, miR-214 regulatory action of *SNCA* mRNA expression is linked with the mechanisms of neuroprotection in Resveratrol, a potential therapeutic drug treatment in PD (Albani et al., 2010), therefore implying that this miRNA may have been the molecular regulator of interest in the mechanism of the resveratrol-therapy in PD. Another is miR-7 that is highly expressed in the neurons and can also bind directly to the 3′UTR of *SNCA* mRNA (Junn et al., 2009; Doxakis, 2013). miR-7 controls the expression of *SNCA* gene by decreasing the mRNA stability rather than modifying the protein translation rate (Ma et al., 2013). Suppression of *SNCA* gene expression by miR-7 will inhibit proteasome impairment and protect the cells from oxidative stress (Junn et al., 2009). In fact, this neuroprotective of miR-7 against α-synuclein aggregates has been replicated in various experimental conditions to induce cell death, including due to impaired mitochondrial activity (Choi et al., 2014; Fragkouli and Doxakis, 2014) or genetic mutation of A53T in SNCA gene (Fan et al., 2016; Zhou et al., 2016). Despite these neuroprotective actions of miR-7, only one LOPD study actually showed the differentially expressed miR-7 in human samples (Table 1), in which its expression was reduced in LOPD patients at the region of gyri cinguli (Tatura et al., 2016). This is particularly important as these findings may imply that miR-7 regulation of α-synuclein is more complicated than the normal direct-mRNA binding and suppression, seen in *in vitro* cells or experimental animals or it may suggest that miR-7 regulates multiple target genes thus its expression is highly maintained or there are other underlying regulators. This is partially supported by a previous study that showed *SNCA* gene expression can be synergistically suppressed by combination of miR-7 and miR-153 action (Doxakis, 2010; Kim et al., 2013; Fragkouli and Doxakis, 2014; Lim and Song, 2014), in which endogenous α-synuclein protein level was 30–40% reduced (Doxakis, 2010). Like miR-7, miR-153 is also highly expressed in the brain and can directly bind to the 3′UTR of *SNCA* mRNA (Doxakis, 2010; Kim et al., 2013; Lim and Song, 2014). This combined regulatory action of miR-7 and miR-153 may therefore explain the lack of alteration in miR-7 and miR-153 expression in PD patients. However, none of these microRNAs are specific enough to differentiate the EOPD from LOPD patients as both miR-34b and c were also altered in LOPD patients (Table 1). The fact that no association of *SNCA* gene was observed in the susceptibility of EOPD (Martin et al., 2011; Schulte and Gasser, 2011) and this fate is also shared by their regulatory miRNAs, these may imply that measuring *SNCA* gene expression may not suitable for early diagnosis of PD. Nevertheless, some these miRNAs were significantly altered in EOPD patients even with no symptoms observed therefore they offer some potential as biomarkers to differentiate EOPD. Further information is needed to explore and identify more regulatory biomarkers in EOPD, as the current studies were limited in numbers (Table 1).

The role of α-synuclein in the brain is currently unclear thus investigating on other miRNAs that indirectly regulate *SNCA* gene expression and α-synuclein protein may provide additional information. Intriguingly, lysosomal-associated membrane protein 2A (LAMP2A) and heat shock protein family A member 8 (HSPA8) can also regulate α-synuclein protein level via chaperone-mediated autophagy (Vogiatzi et al., 2008; Alvarez-Erviti et al., 2010). Thus, few miRNAs like miR-21, miR-224, miR-379, and miR-373 that regulate *LAMP2A* expression (Alvarez-Erviti et al., 2013; Su et al., 2016) and miR-26b, miR-106a, miR-301b, miR-16-1, and miR-320a that regulate *HSPA8* expression (Alvarez-Erviti et al., 2013; Li G. et al., 2014; Zhang and Cheng, 2014) could also be considered as potential biomarkers in PD progression, and these were consistent with their altered expressions in LOPD studies (Table 1). Similar indirect regulation of *SNCA* gene expression by other miRNAs has also been observed, including miR-133b via RAS homolog family member A (RHOA) (Lu X. C. et al., 2015; Niu et al., 2016), miR-433 via fibroblast growth factor 20 (FGF20) (Wang et al., 2008), miR-128 (Decressac et al., 2013), and miR-155 (Thome et al., 2016), which resulted in changes of *SNCA* expression and protein level. Given that some of these microRNAs were found to be significantly altered in LOPD studies (Table 1), thus this neuroprotection of miRNAs network in regulating α-synuclein protein level may therefore potentially be used as biomarker for PD progression, though their roles in differentiating EOPD would requires more investigations, as there was no comparison of EOPD in those previous LOPD studies (Table 1).

Besides α-synuclein, other proteins in the brain have been identified to cause PD, with evidence of dysregulation of protein quality and control system. One such is the *LRRK2* gene that is a candidate gene in autosomal dominant PD (Lesage and Brice, 2009; Kett and Dauer, 2012). LRRK2 is primarily involved in the regulation of neurite maintenance and neuronal survival (MacLeod et al., 2006). Previous study was done in rats to investigate the relationship between *LRRK2* and α-synuclein found that the increase of striatal α-synuclein protein level also increased the mRNA level of *LRRK2* (Gehrke et al., 2010). In fact, regardless of the mutational status of *LRRK2* gene in PD patients with or without LB pathology, ~20–100% of α-synuclein-positive LB also contained high levels of *LRRK2* mRNA (Daher et al., 2014), which suggests that *LRRK2* and α-synuclein have synergetic effects (Guerreiro et al., 2013). Besides that, a soluble microtubule-associated protein tau (MAPT) that modulates the stability of axonal microtubules (Greggio et al., 2006) had been shown to play a role in the PD progression by interacting with LRRK2. Analogous to the amyloid formation by α-synuclein, MAPT can also form aggregates which deposit into neurofibrillary tangles (Li J.-Q. et al., 2014). There was a significant elevation of phosphorylated MAPT at Thr181 and Ser396 locations in *LRRK2*-overexpressing cells (Kawakami et al., 2014). This increase in MAPT phosphorylation was reduced by *LRRK2* knockdown only at position Thr181, suggesting that LRRK2 positively regulates MAPT phosphorylation at the Thr181 location only. While at the Ser396 location, the MAPT phosphorylation was induced by LRRK2 protein due to unknown mechanism (Li J.-Q. et al., 2014). Nevertheless, these phosphorylated MAPT proteins are implicated in PD progression, especially in the role of toxic protein accumulation. Consequently, overexpression of *LRRK2* expression led to reduced locomotor activity and loss of DA (Saha et al., 2009; Serafin et al., 2014), suggesting that LRRK2 plays an important role in conferring early susceptibility to PD, particularly in controlling the protein levels. Like LRRK2, GBA protein which is a lysosomal membrane protein that cleaves the glycosylceramide (Winfield et al., 1997) also plays a role in protein control system. The loss of lysosomal function arising from dysregulation of GBA will lead to cell toxicity due to accumulation of toxic proteins that can contribute to PD progression (Goker-Alpan et al., 2004). A pilot study on the Jewish population, found that N370S, L444P, T369M, and R469H mutations in the *GBA* gene were associated with PD (Clark et al., 2005), though these findings have not been replicated in other populations. Further studies are needed to confirm the role of GBA in progression of PD, particularly in EOPD, as this gene is currently been associated with LOPD susceptibility (Martin et al., 2011; Schulte and Gasser, 2011). Similar to GBA, ubiquitin-proteasome system (UPS) that comprises of three enzymes, E1, E2 and E3 also been implicated in PD (McNaught et al., 2002). This is supported by the role of PRKN, which has E3 ligase function and loss of PRKN activity caused DA neuron loss without formation of LB (Cook et al., 2012). However, unlike GBA, several mutations in *PRKN* gene have been associated with EOPD (Deng et al., 2008; Chen H. et al., 2016), which exclusively indicates that PRKN plays important roles in early events of PD, particularly in EOPD progression.

Several miRNAs have been shown to regulate *LRRK2* and *GBA* expression (Table 1, Figure 1). A previous study of PD patients compared to healthy controls showed that the circulating levels of miR-335-3p, miR-561-3p, and miR-579-3p were associated with PD susceptibility, with miR-561-3p having the strongest association (Saha et al., 2009). In fact, in comparison between LOPD patients and normal controls also revealed that miR-335-3p, miR-561-3p, and miR-579-3p were reduced in whole blood (Yilmaz et al., 2016) and PBMCs (Martins et al., 2011) samples. However, these miRNAs were only predicted to target *LRRK2* gene (Saha et al., 2009), with no evidence of direct regulation. The only confirmed regulatory miRNAs for LRRK2 is miR-205 that can directly bind to 3′UTR region of *LRRK2* mRNA and this miRNA was down-regulated in LOPD patients (Cho et al., 2013). Interestingly, miR-205 prevented neurite outgrowth defects in neurons expressing PD-related *LRRK2* R1441G mutants (Cho et al., 2013), thus suggesting that this miRNA can protect the progression of PD due to LRRK2 genetic susceptibility, which may be a potential therapeutic approach in *LRRK2* PD patients. Another study that compared LRRK2-mutated PD patients to healthy controls (Botta-Orfila et al., 2014), has identified three differentially expressed miRNAs (miR-29c, miR-29a, and miR-19a) in LRRK2-mutated PD patients. Among these miRNAs, two of them (miR-29c and miR-29a) were also found to be differentially expressed in idiopathic PD. As both *LRRK2* PD and idiopathic PD shared similar molecular pathways dysregulation (Botta-Orfila et al., 2014), it implied that the *LRRK2* gene may play a role in early onset of the disease. As for the *MAPT* gene, miR-34c have been shown to directly regulate MAPT phosphorylation and expression yet this was only shown in gastric cancer (Wu H. et al., 2013). Indirect regulation of MAPT phosphorylation was also identified, in which miR-26b regulates RBI (Absalon et al., 2013) and miR-138 regulates RARA/GSK-3β pathway (Wang X. et al., 2015), that resulted in suppression of MAPT phosphorylation. As for *GBA* gene, miR-16–5p, and miR-195–5p are predicted to regulate *GBA* expression, yet only miR-127-5p was shown to reduce GBA activity and protein synthesis (Siebert et al., 2014), and may contribute to neuronal cell toxicity and PD progression. This is in agreement with previous findings as miR-127-5p expression was increased in the brain tissue of LOPD patients (Hoss et al., 2016), consistent with the findings that GBA gene is altered in LOPD patients and certain mutations in *GBA* were tended to be associated with EOPD (Martin et al., 2011; Schulte and Gasser, 2011). However, to what extend that *LRRK2* and *GBA* genes and their regulatory miRNAs can be used to differentiate and identify EOPD in the patients is still unclear, but they surely are reasonable biomarkers for LOPD patients.

### MicroRNAs in oxidative stress

Another implicated pathway in PD is the oxidative stress that plays important role in disease progression including (1) increasing DNA and mitochondrial DNA mutation, (2) dysregulation of protein homeostasis/degradation, (3) regulation of dopamine release, and (4) disruption of cellular self-defense, protection and survival (Dias et al., 2013). Experimental study of PD showed that increased ROS production and alteration of antioxidants were observed (Cassarino et al., 1997). In fact, endogenous antioxidant, the nuclear factor erythroid 2-related factor 2 (NFE2L2) significantly translocate from the cytoplasm into the nucleus to activate antioxidant activity genes in PD (Ramsey et al., 2007). Usually, NFE2L2 is primarily been suppressed by Kelch-like ECH-associated protein 1 (KEAP1, a NFE2L2 sequester protein) in cytoplasm, but upon under oxidative stress, NFE2L2 activates the expression NADPH quinine oxidoreductase 1, heme oxygenase-1, superoxide dismutase, glutathione and many other antioxidant agents/proteins (Satoh et al., 2006; Zhang et al., 2013). Independent of KEAP1 regulation, *NFE2L2* expression can also be regulated by protein kinase C and glycogen synthase kinase 3 beta or epigenetic factors (Bryan et al., 2013). Importantly, *NFE2L2* gene and its genetic variants have been implicated as a possible marker for EOPD in Australian PD study (Todorovic et al., 2015), thus confirming the role of oxidative stress in EOPD progression. Another important molecule in oxidative stress is PARK7 that can stabilize NFE2L2 and able to regulate superoxide dismutase 1 via interaction with ERK1/2-ELK1 pathway (Wang et al., 2011). Previous studies also showed that PARK7 also acts as an antioxidant (Taira et al., 2004) and as a redox-sensor protein to prevent α-synuclein protein aggregation (Abou-Sleiman et al., 2003; Shendelman et al., 2004). PARK7 is proposed to be involved in mitochondrial protection by directly inhibits 20S proteasome activity and recruits NADPH quinone oxidoreductase-1 to co-regulate 20S proteasome activity via stabilization of NFE2L2 (Moscovitz et al., 2015). Similarly to NFE2L2, hemochromatosis gene is also been considered as a potential biomarker for EOPD (Bartzokis et al., 2004), due to its role in iron metabolism and iron-redox balance in oxidative stress (Faucheux et al., 2003). However, recent studies did not find strong relationship of this gene variants, C282Y and H63D with PD in various populations (Aamodt et al., 2007; Duan et al., 2015; Xia et al., 2015), therefore may imply that the role of this hemochromatosis gene in PD may require further investigation.

Several microRNAs that were identified as biomarkers in PD patients are also implicated in oxidative stress via the genes above (Figure 1). One such is miR-153 that can directly regulates *NFE2L2* expression and overexpression of miR-153 led to increase of ROS production in the cells (Narasimhan et al., 2014; Yang W. et al., 2015). This is particularly relevant, as miR-153 expression was increased in CSF of LOPD patients (Gui et al., 2015), which indicates that there are dysregulation of oxidative stress and antioxidant system, consistent with miR-153 regulatory role on NFE2L2. Besides miR-153, miR-7 can also regulate NFE2L2 pathway via direct binding to 3′UTR of *KEAP1* mRNA (Kabaria et al., 2015a). Similarly, miR-141 also regulates *KEAP1* expression (Shi et al., 2015; Wang et al., 2016; Cheng et al., 2017), whereas miR-28 directly regulates *NFE2L2* mRNA, independently of KEAP1 (Yang et al., 2011), but these relationships were only observed in other cell types. Aside from that, miR-494 was identified before can directly downregulate *PARK7* expression by binding to the 3′UTR of *PARK7* mRNA and overexpression of miR-494 reduced PARK7 protein levels and resulted in increased susceptibility of the neuron cells to oxidative stress (Xiong et al., 2014). Consequently, ROS production was increased when miR-494 is overexpressed in neuron cells, and this effect was diminished by an introduction of PARK7 into those miR-494 overexpressed cells (Xiong et al., 2014), confirming the negative regulation of *PARK7* expression by miR-494 that contributes to PD progression. However, expression of miR-494 was not changed in PD patients, regardless of EOPD or LOPD comparison (Table 1).

### MicroRNAs in neuro-inflammation

In relation with oxidative stress, neuro-inflammation and immune response are also implicated as key players in the onset of disease progression for both sporadic and familial PD (Chao et al., 2014; Tiwari and Pal, 2017). In fact, previous studies showed that microglial-mediated inflammatory event happens at the early onset of PD (Ouchi et al., 2005; Iannaccone et al., 2013). In the brain, microglia are the major resident immune cells that provide innate immunity, together with astrocytes and oligodendrocytes (Taylor et al., 2013). Usually, microglia maintains the brain healthy environment with neurotrophic factors like brain-derived neurotrophic factor (BDNF), insulin-like growth factor-1 and interleukin 10 (Taylor et al., 2013). Though, the introduction of toxic pesticides or protein aggregates lead to activation of NFκB pathway (Lee, 2013) and increased neurotoxic factors like inflammatory cytokines (TNFα, tumor necrosis factor alpha, interleukins like IL-1b), chemokines (MCP-1 and CCL2), prostaglandins and pattern recognition receptors like toll-like-receptors and nod-like-receptor (NLRs) (Taylor et al., 2013). Subsequently, uncontrolled inflammation events lead to increase of ROS and oxidative stress and eventually neuronal damage (Taylor et al., 2013). The role of these cytokines and inflammatory factors in PD progression have been discussed recently (Tiwari and Pal, 2017). Among them, NLR family, pyrin domain-containing 1 (NLRP1), 3 (NLRP3) and 5 (NLRP5) are known to contribute to neuroinflammation and neuronal death (Meng et al., 2014; Lawana et al., 2017). Others like alpha-2 macroglobulin (A2M) protein, which is a major component of the brain innate immune system and has been linked with EOPD (Krüger et al., 2000). However, there are studies that shown an opposite relationship and no correlation between A2M and the age-onset of PD (Nicoletti et al., 2002; Tang et al., 2002; Guo et al., 2016). Taken together, these findings suggest that an active inflammatory process in the CNS of PD patients includes innate immune system.

Regulation of inflammatory initiators by microRNAs may provide beneficial outcomes in PD. This is evident by a study of intracerebral hemorrhage in mice revealed that miR-223 can reduce brain edema and inflammation by direct-inhibition of NLRP3 inflammasome (Yang Z. et al., 2015). As miR-223 expression was increased in LOPD patients (Vallelunga et al., 2014), this may indicate that the increase of miR-223 expression in late-stage was probably a negative feedback mechanism to control the neuroinflammation and possibly to reduce cell death in the late-onset of PD. Whereas, miR-7 that also regulates NLRP3 (Zhou et al., 2016) and proto-oncogene, NF-kB subunit/p65 protein (RELA) (Choi et al., 2014), its expression was reduced in the brain tissues of LOPD patients (Tatura et al., 2016) thus indicates a loss of miR-7 neuroprotection in PD progression. In a mice MPTP model of PD, an introduction of miR-7116 prevented TNF-α production and loss of DA neurons, due to miR-7116 can directly bind to TNF-α to inhibit its production (He et al., 2017). Even though, miR-7116 was not implicated with EOPD or LOPD studies (Table 1), yet TNF-α itself can regulates other miRNAs that are known regulators for mitochondrial function and subsequently can contribute to oxidative stress and apoptosis (Prajapati et al., 2015). Thus, miR-7116 may play a greater role in the PD progression, especially in the early stage of inflammatory events. Intriguingly, in EOPD patients, both miR-1, and miR-22 expressions were reduced (Margis et al., 2011), and their common validated target/gene is BDNF (Muiños-Gimeno et al., 2011; Brandenburger et al., 2014; Varendi et al., 2014), which is known to increase dopaminergic neuroprotection (Fumagalli et al., 2006). Therefore, reduced of these microRNAs expression in those EOPD patients may therefore indicates that *BDNF* expression was up-regulated to promote early rescue of DA neurons, thus these miRNAs may potentially be the biomarkers to differentiate EOPD. Despite that, there are other microRNAs that also regulates *BDNF* expression, and some of them are been implicated both in EOPD and LOPD patients (Table 1). Whether these miRNAs can be used to differentiate EOPD specifically enough is unknown and requires further investigations on their sensitivity and specificity.

### MicroRNAs and others genes in parkinson disease

Other pathways than above have also been discussed in the progression of PD (Alonso-Navarro et al., 2014). One such is the drugs and xenobiotics detoxification mechanisms pathways, in which a mice MPTP model of PD study showed that a polymorphism in cytochrome P450 family two subfamily D member 6 (*CYP2D6*) caused susceptibility to PD (Jiménez-Jiménez et al., 1991). Following that, others have shown that some of these detoxification genes, *GSTM1, GSTT1*, and *NAT2* have been implicated in PD with controversial associations (Wang and Wang, 2014; Jiménez-Jiménez et al., 2016a). Importantly, *CYP2D6* and *NAT2* genes have been shown exclusively to be associated with EOPD (Agúndez et al., 1995; Agundez et al., 1998), implying that these detoxification genes would have be suitable as EOPD biomarkers. Similar to that, genes that are involved in dopamine metabolism and transport (*DRD3, SLC6A3, MTHFR*), also showed to pose a risk for PD (Wu Y.-L. et al., 2013; Zhai et al., 2014; Hassan et al., 2016), with particularly, dopamine receptor D3 (*DRD3*) gene that has been implicated in EOPD patients (Hassan et al., 2016). Due to heterogeneity of PD, various other genes have also been associated with PD in one or two populations, including saitohin protein (Lu et al., 2014), vitamin D receptor (Gatto et al., 2016), semaphorin 5A (Yu et al., 2014), granulin (Chen Y. et al., 2016), histamine N-methyltransferase (*HNMT*) (Jiménez-Jiménez et al., 2016b), Ras like without CAAX 2 (Lu Y. et al., 2015; Foo et al., 2017), syntaxin 1B, parkinson disease 16, *FGF20*, glycoprotein nmb (International Parkinson's Disease Genomics and Wellcome Trust Case Control, 2011), serine/threonine kinase 39 (Foo et al., 2017) and huntingtin interacting protein 1 related (*HIP1R*) (Yu et al., 2015) genes. Among these genes, *HNMT* and *HIP1R* genes have been found to be implicated with the age-onset of the disease, specifically for *HNMT* in EOPD (Yang X. et al., 2015) whereas *HIP1R* gene in LOPD (Yu et al., 2015). The roles of these genes in PD progression require further investigation, as some of these genes were only reported once in association, while others have controversial relationships with PD.

Among those miRNAs that been investigated in EOPD and LOPD patients, some of them were found to regulate *CYP2D6* and *DRD3* genes (Table 1, Figure 1). One such is in the comparison between LOPD patients and normal controls, miR-128 expression in CSF samples was found to be reduced (Burgos et al., 2014) and miR-128 can directly bind to *CYP2D6* mRNA and suppression its expression (Li et al., 2015), which may indicates that *CYP2D6* expression was up-regulated in the LOPD patients, consistent with the needs of CYP2D6 to metabolize the neurotoxins in PD. Although Let-7d was no listed in the differentially expressed microRNAs in EOPD and LOPD studies (Table 1), Let-7d was shown to directly regulate the expression of *DRD3* (Zhang et al., 2016), which is also a gene that have been associated with EOPD (Hassan et al., 2016). However, no validated and significant microRNA was found to regulate *HNMT* and *HIP1R* expression in brain specifically. Despite CYP2D6 gene was associated with EOPD (Agúndez et al., 1995; Agundez et al., 1998) yet, its regulatory miRNA was only found to be significant in LOPD studies. The reason for such discrepancies may due to the fact that those LOPD studies did not include a comparison to EOPD patients thus undermines the searching of those miRNAs as biomarkers in EOPD. Also, the possibility of indirect and underlying regulation of these microRNAs and validated targets/genes may also contribute to the inconsistencies. Nevertheless, these miRNAs and their target genes may offer potential diagnostic values in EOPD, as they are significantly altered in patients and some of their target genes are consistently been involved in PD progression, yet further investigations are required to validate such roles.

## Challenges of using miRNA as biomarkers for diagnosis of EOPD

miRNAs have an immense potential as biomarkers for the early diagnosis of PD as they are easily detectable in body fluids and a good number of them correlates with disease progression. Developing a technique to detect PD earlier has become important due to the burden of the disease in which most of the cases are detected when 70–80% of the dopaminergic neurons are lost (Goldenberg, 1990), thus reducing the efficiency of the treatment and recovery. The idea for an early diagnosis to prevent the neuro-degeneration by a simple blood sampling is enticing, and this would make the diagnosis of PD more efficient and economical (Khoo et al., 2012). Currently, the physicians diagnose PD by carefully taking the patient's neurological history and performing a thorough physical examination to detect for the symptoms and signs of PD (Jankovic, 2008). To date, the most accurate testing currently available for PD is to image the dopamine system and brain metabolism through specialized brain scanning, and these tests could only be performed in specialized imaging centers, therefore can be very expensive (Lees et al., 2009). Thus, using the miRNAs as blood biomarkers or CSF sampling give new strategy to improve the screening and diagnosis of PD. In fact, combination of the genetic mutation (DNA biomarker) together with regulatory miRNAs (stable RNA biomarker) in a screening panel could offer a better diagnostic approach, with an ability to differentiate the onset and progression of PD as well-determine their severity, hence a sensitive molecular testing.

One of the problems in identifying microRNAs as biomarkers for any disease is the inconsistency of expression between circulating microRNA and the tissue expression. There are a few explanations behind this situation. One reason is that changes in the concentrations of ubiquitous miRNAs causing or associated with the pathology of the disease can be limited and do not necessarily reflect their concentrations in the body fluids due to the small amount of miRNAs from the affected organ or tissues spilling into the circulatory system (Sheinerman and Umansky, 2013). Another reason is that the changes in miRNA expression are more prominent at the site of the pathological hallmark for a particular disease (PD: in substantia nigra) (Heman-Ackah et al., 2013). For a disease like PD, due to blood brain barrier, miRNAs from the brain that are detectable in the serum or plasma samples may be very limited (Sheinerman and Umansky, 2013), hence may not reflect the miRNA expression in the tissue. Therefore, identification of these miRNAs for a purpose of marking each step of disease progression would therefore require more extensive and detailed studies.

It is also a huge challenge to pick the ultimate miRNAs as sensitive biomarkers for EOPD patients. Previous studies in EOPD patients (Table 1) revealed that there were about 10 significantly expressed miRNAs in EOPD patients, yet their expression was similarly reduced in both EOPD and LOPD patients, except for miR-1, miR-22, and miR-331-5p. Even with that, miR-1 and miR-22 expressions were also found to be reduced in brain tissue (Liao et al., 2013) and in CSF (Gui et al., 2015) samples of LOPD patients, thus indicates that their expressions are also not specific enough for the detection of EOPD. Therefore, we have only one miRNA, miR-331-5p, in which its expression was increased in EOPD patients (Cardo et al., 2013). Focusing on the brain studies, there is one gene target of miR-331 which is the neuropilin 2 (NRP2) that is shown to promote the cell growth and proliferation of glioblastoma (Epis et al., 2014), yet the role of this gene in PD is unknown. Moreover, miR-331 was implicated in neuroprotection in ischemic cortex (Hunsberger et al., 2012) and therefore may suggest that this miRNA possess significant roles in PD but its function requires further study. The fact that its expression was increased in EOPD (Cardo et al., 2013) and never been implicated in LOPD studies, thus these findings imply that miR-331 has a strong potential as a biomarker for EOPD, though further information and studies are needed to validate its role and relationship in PD. Since the published EOPD studies was in lacking in numbers when compared to LOPD patients (Table 1), there is a need to explore more miRNA profile in EOPD and compared to LOPD patients. Taken together of these findings in PD, it is clear that early events of PD progression, particularly in mitochondrial dysfunction, oxidative stress and neuroinflammation may hold the key genes and miRNAs for EOPD diagnosis. Furthermore, from the known mutations in PD, PRKN, PINK1, NFE2L2, A2M, and PARK7 genes (mitochondrial, oxidative genes and neuro-inflammation) (Krüger et al., 2000; Klein and Westenberger, 2012; Lin and Farrer, 2014; Todorovic et al., 2015) and other genes like DRD3 (Hassan et al., 2016) and HNMT (Yang X. et al., 2015) were showed to be associated with EOPD. Therefore, confirming the roles of these genes in early events of PD progression. So, further investigation are needed to allow for new identification of genes and their regulatory miRNAs thus consequently to find new biomarker for PD. It is also worth to mention that the miRNA-based research in PD is still in early exploring stages and not fully clear yet. Even though there are few discrepancies in the findings and its biological targets, some of these miRNAs showed potentials as biomarkers of EOPD, but the limitations in the amount of the previous published studies as well as the PD heterogeneity may therefore emphasize on the need for further investigations and research.

## Conclusion

The characterization and differentiation of EOPD from LOPD may reveal important mechanisms for PD susceptibility and can help tailor the effective management of each subtype. As miRNAs are shown to regulate important genes such as *PRKN, PARK7, PINK1, SNCA*, and others genes in early event of PD progression, they could potentially be harnessed as biomarkers to diagnose EOPD and possibly to improve the management of PD. Although the idea of miRNA-based biomarkers is tempting, few limitations are needed to be considered. Majority of these miRNAs were found in LOPD studies, and the lack of the comparison to EOPD patients may undermine the actual progression marker by miRNA detection. Second, few of the significantly miRNAs were inconsistently expressed between samples types and studies and some of them were not consistently detected either. Therefore, by selecting the miRNAs in comparison of their targets/genes could provide further clarifications on their roles in diagnosing EOPD. Nevertheless, to what extent these miRNAs can be potentially used as biomarkers for early diagnosis for PD is unknown, and would therefore need extensive studies to characterize their regulatory and functional outcomes in differentiating subsets of PD.

## Author contributions

AA drafted and wrote this manuscript, AS and SS were responsible for manuscript writing, editing and critical evaluation, NA, RJ, and NM were responsible for idea conception, critical evaluation and manuscript review.

### Conflict of interest statement

The authors declare that the research was conducted in the absence of any commercial or financial relationships that could be construed as a potential conflict of interest.

## References

[B1] Aamodt A. H. Stovner L. J. Thorstensen K. Lydersen S. White L. R. Aasly J. O. (2007). Prevalence of haemochromatosis gene mutations in Parkinson's disease. J. Neurol. Neurosur. Psychiatry 78, 315–317. 10.1136/jnnp.2006.101352 17056630 PMC2117639

[B2] Abou-Sleiman P. M. Healy D. G. Quinn N. Lees A. J. Wood N. W. (2003). The role of pathogenic DJ-1 mutations in Parkinson's disease. Ann. Neurol. 54, 283–286. 10.1002/ana.10675 12953260

[B3] Absalon S. Kochanek D. M. Raghavan V. Krichevsky A. M. (2013). MiR-26b, Upregulated in Alzheimer's disease, activates cell cycle entry, tau-phosphorylation, and apoptosis in postmitotic neurons. J. Neurosci. 33, 14645–14659. 10.1523/JNEUROSCI.1327-13.2013 24027266 PMC3810537

[B4] Agúndez J. A. G. Jiménez-Jiménez F. J. Luengo A. Bernal M. L. Molina J. A. Ayuso L.. (1995). Association between the oxidative polymorphism and early onset of Parkinson's disease. Clin. Pharmacol. Ther. 57, 291–298. 10.1016/0009-9236(95)90154-X 7697946

[B5] Agundez J. A. Jimenez-Jimenez F. J. Luengo A. Molina J. A. Orti-Pareja M. Vazquez A.. (1998). Slow allotypic variants of the NAT2 gene and susceptibility to early-onset Parkinson's disease. Neurology 51, 1587–1592. 10.1212/WNL.51.6.1587 9855506

[B6] Albani D. Polito L. Signorini A. Forloni G. (2010). Neuroprotective properties of resveratrol in different neurodegenerative disorders. Biofactors 36, 370–376. 10.1002/biof.118 20848560

[B7] Alonso-Navarro H. Jimenez-Jimenez F. J. Garcia-Martin E. Agundez J. A. (2014). Genomic and pharmacogenomic biomarkers of Parkinson's disease. Curr. Drug Metab. 15, 129–181. 10.2174/138920021502140327175404 24694231

[B8] Alvarez-Erviti L. Rodriguez-Oroz M. C. Cooper J. M. Caballero C. Ferrer I. Obeso J. A.. (2010). Chaperone-mediated autophagy markers in parkinson disease brains. Arch. Neurol. 67, 1464–1472. 10.1001/archneurol.2010.198 20697033

[B9] Alvarez-Erviti L. Seow Y. Schapira A. H. Rodriguez-Oroz M. C. Obeso J. A. Cooper J. M. (2013). Influence of microRNA deregulation on chaperone-mediated autophagy and α-synuclein pathology in Parkinson's disease. Cell Death Dis. 4:e545. 10.1038/cddis.2013.73 23492776 PMC3615743

[B10] Ascherio A. Schwarzschild M. A. (2016). The epidemiology of Parkinson's disease: risk factors and prevention. Lancet Neurol. 15, 1257–1272. 10.1016/S1474-4422(16)30230-7 27751556

[B11] Ba F. Martin W. R. (2015). Dopamine transporter imaging as a diagnostic tool for parkinsonism and related disorders in clinical practice. Parkinsonism Relat. Disord. 21, 87–94. 10.1016/j.parkreldis.2014.11.007 25487733

[B12] Bai X. Tang Y. Yu M. Wu L. Liu F. Ni J.. (2017). Downregulation of blood serum microRNA 29 family in patients with Parkinson's disease. Sci. Rep. 7:5411. 10.1038/s41598-017-03887-3 28710399 PMC5511199

[B13] Bartel D. P. (2004). MicroRNAs: genomics, biogenesis, mechanism, and function. Cell 116, 281–297. 10.1016/S0092-8674(04)00045-5 14744438

[B14] Bartzokis G. Tishler T. A. Shin I.-S. Lu P. H. Cummings J. L. (2004). Brain ferritin iron as a risk factor for age at onset in neurodegenerative diseases. Ann. N. Y. Acad. Sci. 1012, 224–236. 10.1196/annals.1306.019 15105269

[B15] Batistela M. S. Josviak N. D. Sulzbach C. D. de Souza R. L. (2017). An overview of circulating cell-free microRNAs as putative biomarkers in Alzheimer's and Parkinson's Diseases. Int. J. Neurosci. 127, 547–558. 10.1080/00207454.2016.1209754 27381850

[B16] Bekris L. M. Mata I. F. Zabetian C. P. (2010). The genetics of Parkinson Disease. J. Geriat. Psychiatry Neurol. 23, 228–242. 10.1177/0891988710383572 20938043 PMC3044594

[B17] Bonifati V. Rohe C. F. Breedveld G. J. Fabrizio E. De Mari M. Tassorelli C.. (2005). Early-onset parkinsonism associated with PINK1 mutations: frequency, genotypes, and phenotypes. Neurology 65, 87–95. 10.1212/01.wnl.0000167546.39375.82 16009891

[B18] Botta-Orfila T. Morat,ó X. Compta Y. Lozano J. J. Falgàs N. Valldeoriola F.. (2014). Identification of blood serum micro-RNAs associated with idiopathic and LRRK2 Parkinson's disease. J. Neurosci. Res. 92, 1071–1077. 10.1002/jnr.23377 24648008

[B19] Braak H. Rüb U. Gai W. P. Del Tredici K. (2003). Idiopathic Parkinson's disease: possible routes by which vulnerable neuronal types may be subject to neuroinvasion by an unknown pathogen. J. Neural Transm. 110, 517–536. 10.1007/s00702-002-0808-2 12721813

[B20] Brandenburger T. Grievink H. Heinen N. Barthel F. Huhn R. Stachuletz F.. (2014). Effects of remote ischemic preconditioning and myocardial ischemia on microRNA-1 expression in the rat heart *in vivo*. Shock 42, 234–238. 10.1097/SHK.0000000000000201 24978894

[B21] Bryan H. K. Olayanju A. Goldring C. E. Park B. K. (2013). The Nrf2 cell defence pathway: Keap1-dependent and -independent mechanisms of regulation. Biochem. Pharmacol. 85, 705–717. 10.1016/j.bcp.2012.11.016 23219527

[B22] Burgos K. Malenica I. Metpally R. Courtright A. Rakela B. Beach T.. (2014). Profiles of extracellular miRNA in cerebrospinal fluid and serum from patients with Alzheimer's and Parkinson's diseases correlate with disease status and features of pathology. PLoS ONE 9:e94839. 10.1371/journal.pone.0094839 24797360 PMC4010405

[B23] Caputo V. Sinibaldi L. Fiorentino A. Parisi C. Catalanotto C. Pasini A.. (2011). Brain Derived Neurotrophic Factor (BDNF) expression is regulated by microRNAs miR-26a and miR-26b allele-specific binding. PLoS ONE 6:e28656. 10.1371/journal.pone.0028656 22194877 PMC3237476

[B24] Cardo L. F. Coto E. de Mena L. Ribacoba R. Moris G. Menéndez M.. (2013). Profile of microRNAs in the plasma of Parkinson's disease patients and healthy controls. J. Neurol. 260, 1420–1422. 10.1007/s00415-013-6900-8 23543376

[B25] Cardo L. F. Coto E. Ribacoba R. Menéndez M. Moris G. Suárez E.. (2014). MiRNA profile in the substantia nigra of parkinson's disease and healthy subjects. J. Mol. Neurosci. 54, 830–836. 10.1007/s12031-014-0428-y 25284245

[B26] Cassarino D. S. Fall C. P. Swerdlow R. H. Smith T. S. Halvorsen E. M. Miller S. W.. (1997). Elevated reactive oxygen species and antioxidant enzyme activities in animal and cellular models of Parkinson's disease. Biochim. Biophys. Acta 1362, 77–86. 10.1016/S0925-4439(97)00070-7 9434102

[B27] Chao Y. Wong S. C. Tan E. K. (2014). Evidence of inflammatory system involvement in Parkinson's Disease. Biomed Res. Int. 2014:308654. 10.1155/2014/308654 25050341 PMC4094726

[B28] Chaudhuri A. D. Choi D. C. Kabaria S. Tran A. Junn E. (2016). MicroRNA-7 regulates the function of mitochondrial permeability transition pore by targeting VDAC1 expression. J. Biol. Chem. 291, 6483–6493. 10.1074/jbc.M115.691352 26801612 PMC4813563

[B29] Chen H. Huang X. Yuan L. Xia H. Xu H. Yang Y.. (2016). A homozygous parkin p.G284R mutation in a Chinese family with autosomal recessive juvenile parkinsonism. Neurosci. Lett. 624, 100–104. 10.1016/j.neulet.2016.05.011 27177722

[B30] Chen Y. Cao B. Ou R. Chen X. Zhao B. Wei Q.. (2016). Association analysis of the GRN rs5848 and MAPT rs242557 polymorphisms in Parkinson's disease and multiple system atrophy: a large-scale population-based study and meta-analysis. Int. J. Neurosci. 126, 947–954. 10.3109/00207454.2015.1086345 26303052

[B31] Cheng L.-B. Li K.-R. Yi N. Li X.-M. Wang F. Xue B.. (2017). miRNA-141 attenuates UV-induced oxidative stress via activating Keap1-Nrf2 signaling in human retinal pigment epithelium cells and retinal ganglion cells. Oncotarget 8, 13186–13194. 10.18632/oncotarget.14489 28061435 PMC5355087

[B32] Cheng M. Liu L. Lao Y. Liao W. Liao M. Luo X.. (2016). MicroRNA-181a suppresses parkin-mediated mitophagy and sensitizes neuroblastoma cells to mitochondrial uncoupler-induced apoptosis. Oncotarget 7, 42274–42287. 10.18632/oncotarget.9786 27281615 PMC5173134

[B33] Cho H. J. Liu G. Jin S. M. Parisiadou L. Xie C. Yu J.. (2013). MicroRNA-205 regulates the expression of Parkinson's disease-related leucine-rich repeat kinase 2 protein. Human Mol. Genet. 22, 608–620. 10.1093/hmg/dds470 23125283 PMC3542867

[B34] Choi D. C. Chae Y.-J. Kabaria S. Chaudhuri A. D. Jain M. R. Li H.. (2014). MicroRNA-7 protects against 1-methyl-4-phenylpyridinium-induced cell death by targeting RelA. J. Neurosci. 34, 12725–12737. 10.1523/JNEUROSCI.0985-14.2014 25232110 PMC4166159

[B35] Choy F. C. Klarić T. S. Koblar S. A. Lewis M. D. (2017). miR-744 and miR-224 downregulate Npas4 and affect lineage differentiation potential and neurite development during neural differentiation of mouse embryonic stem cells. Mol. Neurobiol. 54, 3528–3541. 10.1007/s12035-016-9912-4 27189618

[B36] Cimmino A. Calin G. A. Fabbri M. Iorio M. V. Ferracin M. Shimizu M.. (2005). miR-15 and miR-16 induce apoptosis by targeting BCL2. Proc. Natl. Acad. Sci. U.S.A. 102, 13944–13949. 10.1073/pnas.0506654102 16166262 PMC1236577

[B37] Clark L. N. Nicolai A. Afridi S. Harris J. Mejia-Santana H. Strug L.. (2005). Pilot association study of the β-glucocerebrosidase N370S allele and Parkinson's disease in subjects of Jewish ethnicity. Mov. Disord. 20, 100–103. 10.1002/mds.20320 15517591

[B38] Cook C. Stetler C. Petrucelli L. (2012). Disruption of protein quality control in Parkinson's Disease. Cold Spring Harb. Perspect. Med. 2:a009423. 10.1101/cshperspect.a009423 22553500 PMC3331692

[B39] Daher J. P. L. Volpicelli-Daley L. A. Blackburn J. P. Moehle M. S. West A. B. (2014). Abrogation of α-synuclein–mediated dopaminergic neurodegeneration in LRRK2-deficient rats. Proc. Natl. Acad. Sci. U.S.A. 111, 9289–9294. 10.1073/pnas.1403215111 24927544 PMC4078806

[B40] Dauer W. Przedborski S. (2003). Parkinson's Disease. Neuron 39, 889–909. 10.1016/S0896-6273(03)00568-3 12971891

[B41] Dawson T. M. Ko H. S. Dawson V. L. (2010). Genetic animal models of Parkinson's disease. Neuron 66, 646–661. 10.1016/j.neuron.2010.04.034 20547124 PMC2917798

[B42] Deas E. Wood N. W. Plun-Favreau H. (2011). Mitophagy and Parkinson's disease: The PINK1–parkin link(). Biochim. Biophys. Acta 1813, 623–633. 10.1016/j.bbamcr.2010.08.007 20736035 PMC3925795

[B43] Decressac M. Mattsson B. Weikop P. Lundblad M. Jakobsson J. Björklund A. (2013). TFEB-mediated autophagy rescues midbrain dopamine neurons from α-synuclein toxicity. Proc. Natl. Acad. Sci. U.S.A. 110, E1817–E1826. 10.1073/pnas.1305623110 23610405 PMC3651458

[B44] Deng H. Le W. Shahed J. Xie W. Jankovic J. (2008). Mutation analysis of the parkin and PINK1 genes in American Caucasian early-onset Parkinson disease families. Neurosci. Lett. 430, 18–22. 10.1016/j.neulet.2007.10.018 18068301

[B45] Dias V. Junn E. Mouradian M. M. (2013). The role of oxidative stress in Parkinson's Disease. J. Parkinson's Dis. 3, 461–491. 10.3233/JPD-130230 24252804 PMC4135313

[B46] Ding H. Huang Z. Chen M. Wang C. Chen X. Chen J.. (2016). Identification of a panel of five serum miRNAs as a biomarker for Parkinson's disease. Parkinsonism Relat. Disord. 22, 68–73. 10.1016/j.parkreldis.2015.11.014 26631952

[B47] Dong H. Wang C. Lu S. Yu C. Huang L. Feng W.. (2016). A panel of four decreased serum microRNAs as a novel biomarker for early Parkinson's disease. Biomarkers 21, 129–137. 10.3109/1354750X.2015.1118544 26631297

[B48] Dorval V. Mandemakers W. Jolivette F. Coudert L. Mazroui R. De Strooper B.. (2014). Gene and microRNA transcriptome analysis of Parkinson's related LRRK2 mouse models. PLoS ONE 9:e85510. 10.1371/journal.pone.0085510 24427314 PMC3888428

[B49] dos Santos M. C. T. Bell R. da Costa A. N. (2016). Recent developments in circulating biomarkers in Parkinson's disease: the potential use of miRNAs in a clinical setting. Bioanalysis 8, 2497–2518. 10.4155/bio-2016-0166 27855513

[B50] Doxakis E. (2010). Post-transcriptional regulation of α-synuclein expression by mir-7 and mir-153. J. Biol. Chem. 285, 12726–12734. 10.1074/jbc.M109.086827 20106983 PMC2857101

[B51] Doxakis E. (2013). Principles of miRNA-target regulation in Metazoan models. Int. J. Mol. Sci. 14, 16280–16302. 10.3390/ijms140816280 23965954 PMC3759911

[B52] Duan C. Wang M. Zhang Y. Wei X. Huang Y. Zhang H.. (2015). C282Y and H63D polymorphisms in hemochromatosis gene and risk of Parkinson's Disease. Am. J. Alzheimers Dis. Other Demen. 31, 201–207. 10.1177/1533317515602220 26340960 PMC10852941

[B53] Epis M. R. Giles K. M. Candy P. A. Webster R. J. Leedman P. J. (2014). miR-331-3p regulates expression of neuropilin-2 in glioblastoma. J. Neurooncol. 116, 67–75. 10.1007/s11060-013-1271-7 24142150 PMC3889298

[B54] Fan Z. Lu M. Qiao C. Zhou Y. Ding J.-H. Hu G. (2016). MicroRNA-7 enhances subventricular zone neurogenesis by inhibiting NLRP3/Caspase-1 axis in adult neural stem cells. Mol. Neurobiol. 53, 7057–7069. 10.1007/s12035-015-9620-5 26676570

[B55] Farrer M. J. (2006). Genetics of Parkinson disease: paradigm shifts and future prospects. Nat. Rev. Genet. 7, 306–318. 10.1038/nrg1831 16543934

[B56] Faucheux B. A. Martin M.-E. Beaumont C. Hauw J.-J. Agid Y. Hirsch E. C. (2003). Neuromelanin associated redox-active iron is increased in the substantia nigra of patients with Parkinson's disease. J. Neurochem. 86, 1142–1148. 10.1046/j.1471-4159.2003.01923.x 12911622

[B57] Feng J. Y. Huang B. Yang W.-Q. Zhang Y.-H. Wang L. M. Wang L.-J.. (2015). The putaminal abnormalities on 3.0T magnetic resonance imaging: can they separate parkinsonism-predominant multiple system atrophy from Parkinson's disease? Acta Radiol. 56, 322–328. 10.1177/0284185114524090 24619850

[B58] Fereshtehnejad S.-M. Hadizadeh H. Farhadi F. Shahidi G. A. Delbari A. Lökk J. (2014). Comparison of the psychological symptoms and disease-specific quality of life between early- and typical-onset Parkinson's Disease patients. Parkinson's Dis. 2014:7. 10.1155/2014/819260 25614849 PMC4295150

[B59] Ferguson L. W. Rajput A. H. Rajput A. (2015). Early-onset vs. late-onset Parkinson's disease: a clinical-pathological study. Can. J. Neurol. Sci. 43, 113–119. 10.1017/cjn.2015.244 26189779

[B60] Foo J. N. Tan L. C. Irwan I. D. Au W.-L. Low H. Q. Prakash K.-M.. (2017). Genome-wide association study of Parkinson's disease in East Asians. Human Mol. Genet. 26, 226–232. 10.1093/hmg/ddw379 28011712

[B61] Fragkouli A. Doxakis E. (2014). miR-7 and miR-153 protect neurons against MPP(+)-induced cell death via upregulation of mTOR pathway. Front. Cell. Neurosci. 8:182. 10.3389/fncel.2014.00182 25071443 PMC4080263

[B62] Fu Y. Zhen J. Lu Z. (2017). Synergetic neuroprotective effect of docosahexaenoic acid and aspirin in SH-Y5Y by inhibiting miR-21 and activating RXRα and PPARα. DNA Cell Biol. 36, 482–489. 10.1089/dna.2017.3643 28346830

[B63] Fumagalli F. Racagni G. Riva M. A. (2006). Shedding light into the role of BDNF in the pharmacotherapy of Parkinson's disease. Pharmacogenomics J. 6, 95–104. 10.1038/sj.tpj.6500360 16402079

[B64] Gasser T. Hardy J. Mizuno Y. (2011). Milestones in PD genetics. Mov. Disord. 26, 1042–1048. 10.1002/mds.23637 21626549

[B65] Gatto N. M. Paul K. C. Sinsheimer J. S. Bronstein J. M. Bordelon Y. Rausch R.. (2016). Vitamin D receptor gene polymorphisms and cognitive decline in Parkinson's disease. J. Neurol. Sci. 370, 100–106. 10.1016/j.jns.2016.09.013 27772736 PMC5325129

[B66] Gehrke S. Imai Y. Sokol N. Lu B. (2010). Pathogenic LRRK2 negatively regulates microRNA-mediated translational repression. Nature 466, 637–641. 10.1038/nature09191 20671708 PMC3049892

[B67] Goker-Alpan O. Schiffmann R. LaMarca M. E. Nussbaum R. L. McInerney-Leo A. Sidransky E. (2004). Parkinsonism among Gaucher disease carriers. J. Med. Genet. 41, 937–940. 10.1136/jmg.2004.024455 15591280 PMC1735652

[B68] Goldenberg G. (1990). Performance of concurrent non-motor tasks in parkinson's disease. J. Neurol. 237, 191–196. 10.1007/BF00314593 2370567

[B69] Greggio E. Jain S. Kingsbury A. Bandopadhyay R. Lewis P. Kaganovich A.. (2006). Kinase activity is required for the toxic effects of mutant LRRK2/dardarin. Neurobiol. Dis. 23, 329–341. 10.1016/j.nbd.2006.04.001 16750377

[B70] Guerreiro P. S. Huang Y. Gysbers A. Cheng D. Gai W. P. Outeiro T. F.. (2013). LRRK2 interactions with α-synuclein in Parkinson's disease brains and in cell models. J. Mol. Med. (Berl). 91, 513–522. 10.1007/s00109-012-0984-y 23183827 PMC3611031

[B71] Gui Y. Liu H. Zhang L. Lv W. Hu X. (2015). Altered microRNA profiles in cerebrospinal fluid exosome in Parkinson disease and Alzheimer disease. Oncotarget 6, 37043–37053. 10.18632/oncotarget.6158 26497684 PMC4741914

[B72] Guo J.-F. Li K. Yu R.-L. Sun Q.-Y. Wang L. Yao L.-Y.. (2015). Polygenic determinants of Parkinson's disease in a Chinese population. Neurobiol. Aging 36, 1765.e1761–1765.e1766. 10.1016/j.neurobiolaging.2014.12.030 25623333

[B73] Guo X. Tang P. Li X. Chong L. Zhang X. Li R. (2016). Association between two α-2-macroglobulin gene polymorphisms and Parkinson's disease: a meta-analysis. Int. J. Neurosci. 126, 193–198. 10.3109/00207454.2014.996641 25495992

[B74] Gwinn K. David K. K. Swanson-Fischer C. Albin R. Hillaire-Clarke C. S. Sieber B.-A.. (2017). Parkinson's disease biomarkers: perspective from the NINDS Parkinson's Disease biomarkers program. Biomark. Med. 11, 451–473. 10.2217/bmm-2016-0370 28644039 PMC5619098

[B75] Hague S. Rogaeva E. Hernandez D. Gulick C. Singleton A. Hanson M.. (2003). Early-onset Parkinson's disease caused by a compound heterozygous DJ-1 mutation. Ann. Neurol. 54, 271–274. 10.1002/ana.10663 12891685

[B76] Hamza T. H. Payami H. (2010). The heritability of risk and age at onset of Parkinson's disease after accounting for known genetic risk factors. J. Human Genet. 55, 241–243. 10.1038/jhg.2010.13 20203693 PMC2947819

[B77] Hardy J. Lewis P. Revesz T. Lees A. Paisan-Ruiz C. (2009). The genetics of Parkinson's syndromes: a critical review. Curr. Opin. Genet. Dev. 19, 254–265. 10.1016/j.gde.2009.03.008 19419854

[B78] Hassan A. Heckman M. G. Ahlskog J. E. Wszolek Z. K. Serie D. J. Uitti R. J.. (2016). Association of Parkinson disease age of onset with DRD2, DRD3 and GRIN2B polymorphisms. Parkinsonism Relat. Disord. 22, 102–105. 10.1016/j.parkreldis.2015.11.016 26627941 PMC5954834

[B79] Hayashi T. Ishimori C. Takahashi-Niki K. Taira T. Kim Y. C. Maita H.. (2009). DJ-1 binds to mitochondrial complex I and maintains its activity. Biochem. Biophys. Res. Commun. 390, 667–672. 10.1016/j.bbrc.2009.10.025 19822128

[B80] He Q. Wang Q. Yuan C. Wang Y. (2017). Downregulation of miR-7116-5p in microglia by MPP+ sensitizes TNF-α production to induce dopaminergic neuron damage. Glia 65, 1251–1263. 10.1002/glia.23153 28543680

[B81] Healy D. G. Falchi M. O'Sullivan S. S. Bonifati V. Durr A. Bressman S.. (2008). Phenotype, genotype, and worldwide genetic penetrance of LRRK2-associated Parkinson's disease: a case-control study. Lancet Neurol. 7, 583–590. 10.1016/S1474-4422(08)70117-0 18539534 PMC2832754

[B82] Heman-Ackah S. M. Hallegger M. Rao M. S. Wood M. J. (2013). RISC in PD: the impact of microRNAs in Parkinson's disease cellular and molecular pathogenesis. Front. Mol. Neurosci. 6:40. 10.3389/fnmol.2013.00040 24312000 PMC3834244

[B83] Hoss A. G. Labadorf A. Beach T. G. Latourelle J. C. Myers R. H. (2016). microRNA profiles in Parkinson's disease prefrontal cortex. Front. Aging Neurosci. 8:36. 10.3389/fnagi.2016.00036 26973511 PMC4772525

[B84] Hu Q. Wang G. (2016). Mitochondrial dysfunction in Parkinson's disease. Transl. Neurodegeneration 5:14. 10.1186/s40035-016-0060-6 27453777 PMC4957882

[B85] Hunsberger J. G. Fessler E. B. Wang Z. Elkahloun A. G. Chuang D.-M. (2012). Post-insult valproic acid-regulated microRNAs: potential targets for cerebral ischemia. Am. J. Transl. Res. 4, 316–332. 22937209 PMC3426385

[B86] Hwang I. Sohn C.-H. Kang K. M. Jeon B. S. Kim H.-J. Choi S. H.. (2015). Differentiation of parkinsonism-predominant multiple system atrophy from idiopathic Parkinson disease using 3T susceptibility-weighted MR imaging, focusing on putaminal change and lesion asymmetry. Am. J. Neuroradiol. 36, 2227–2234. 10.3174/ajnr.A4442 26338919 PMC7964268

[B87] Iannaccone S. Cerami C. Alessio M. Garibotto V. Panzacchi A. Olivieri S.. (2013). *In vivo* microglia activation in very early dementia with Lewy bodies, comparison with Parkinson's disease. Parkinsonism Relat. Disord. 19, 47–52. 10.1016/j.parkreldis.2012.07.002 22841687

[B88] Inzelberg R. Schecthman E. Paleacu D. Zach L. Bonwitt R. Carasso R. L.. (2004). Onset and progression of disease in familial and sporadic Parkinson's disease. Am. J. Med. Genet. A 124A, 255–258. 10.1002/ajmg.a.20405 14708097

[B89] International Parkinson's Disease Genomics and Wellcome Trust Case Control (2011). A two-stage meta-analysis identifies several new loci for Parkinson's disease. PLoS Genet. 7:e1002142 10.1371/journal.pgen.1002142 21738488 PMC3128098

[B90] Jankovic J. (2008). Parkinson's disease: clinical features and diagnosis. J. Neurol. Neurosurg. Psychiatry 79, 368–376. 10.1136/jnnp.2007.131045 18344392

[B91] Jiménez-Jiménez F. J. Alonso-Navarro H. García-Martín E. Agúndez J. A. (2016a). NAT2 polymorphisms and risk for Parkinson's disease: a systematic review and meta-analysis. Expert Opin. Drug Metab. Toxicol. 12, 937–946. 10.1080/17425255.2016.1192127. 27216438

[B92] Jiménez-Jiménez F. J. Alonso-Navarro H. García-Martín E. Agúndez J. A. (2016b). Thr105Ile (rs11558538) polymorphism in the histamine N-methyltransferase (HNMT) gene and risk for Parkinson disease: a PRISMA-compliant systematic review and meta-analysis. Medicine 95:e4147. 10.1097/MD.0000000000004147 27399132 PMC5058861

[B93] Jiménez-Jiménez F. J. Tabernero C. Mena M. A. García de Yébenes J. Jesús García de Yébenes M. Casarejos M. J.. (1991). Acute effects of 1-Methyl-4-Phenyl-1, 2, 3, 6-tetrahydropyridine in a model of rat designated a poor metabolizer of debrisoquine. J. Neurochem. 57, 81–87. 10.1111/j.1471-4159.1991.tb02102.x 1711101

[B94] Jin S. Dai Y. Li C. Fang X. Han H. Wang D. (2016). MicroRNA-544 inhibits glioma proliferation, invasion and migration but induces cell apoptosis by targeting PARK7. Am. J. Transl. Res. 8, 1826–1837. 27186306 PMC4859911

[B95] Junn E. Lee K.-W. Jeong B. S. Chan T. W. Im J.-Y. Mouradian M. M. (2009). Repression of α-synuclein expression and toxicity by microRNA-7. Proc. Natl. Acad. Sci. U.S.A. 106, 13052–13057. 10.1073/pnas.0906277106 19628698 PMC2722353

[B96] Kabaria S. Choi D. C. Chaudhuri A. D. Jain M. R. Li H. Junn E. (2015a). MicroRNA-7 activates Nrf2 pathway by targeting Keap1 expression. Free Radic. Biol. Med. 89, 548–556. 10.1016/j.freeradbiomed.2015.09.010 26453926 PMC4684759

[B97] Kabaria S. Choi D. C. Chaudhuri A. D. Mouradian M. M. Junn E. (2015b). Inhibition of miR-34b and miR-34c enhances α-synuclein expression in Parkinson's disease. FEBS Lett. 589, 319–325. 10.1016/j.febslet.2014.12.014 25541488 PMC4306645

[B98] Kawakami F. Shimada N. Ohta E. Kagiya G. Kawashima R. Maekawa T.. (2014). Leucine-rich repeat kinase 2 regulates tau phosphorylation through direct activation of glycogen synthase kinase-3β. FEBS J. 281, 3–13. 10.1111/febs.12579 24165324

[B99] Kett L. R. Dauer W. T. (2012). Leucine-rich repeat kinase 2 for beginners: Six key questions. Cold Spring Harb. Perspect. Med. 2:a009407. 10.1101/cshperspect.a009407 22393539 PMC3282500

[B100] Khoo S. K. Petillo D. Kang U. J. Resau J. H. Berryhill B. Linder J.. (2012). Plasma-based circulating MicroRNA biomarkers for Parkinson's disease. J. Parkinsons Dis. 2, 321–331. 10.3233/JPD-012144 23938262

[B101] Kim H.-J. Park G. Jeon B. S. Park W. Y. Kim Y. E. (2013). A mir-153 binding site variation in SNCA in a patient with Parkinson's disease. Mov. Disord. 28, 1755–1756. 10.1002/mds.25505 23674501

[B102] Klein C. Westenberger A. (2012). Genetics of Parkinson's disease. Cold Spring Harb. Perspect. Med. 2:a008888. 10.1101/cshperspect.a008888 22315721 PMC3253033

[B103] Kolata G. (1983). Monkey model of Parkinson's disease. Science 220, 705–705. 6403987 10.1126/science.6403987

[B104] Kraft E. Trenkwalder C. Auer D. P. (2002). T2^*^-weighted MRI differentiates multiple system atrophy from Parkinson's disease. Neurology 59, 1265–1267. 10.1212/01.WNL.0000032757.66992.3C 12391363

[B105] Krüger R. Menezes-Saecker A. M. Schöls L. Kuhn W. Müller T. Woitalla D.. (2000). Genetic analysis of the alpha2-macroglobulin gene in early- and late-onset Parkinson's disease. Neuroreport 11, 2439–2442. 10.1097/00001756-200008030-00020 10943700

[B106] Langston J. W. Ballard P. Tetrud J. W. Irwin I. (1983). Chronic Parkinsonism in humans due to a product of meperidine-analog synthesis. Science 219, 979–980. 6823561 10.1126/science.6823561

[B107] LaVoie M. J. Ostaszewski B. L. Weihofen A. Schlossmacher M. G. Selkoe D. J. (2005). Dopamine covalently modifies and functionally inactivates parkin. Nat. Med. 11, 1214–1221. 10.1038/nm1314 16227987

[B108] Lawana V. Singh N. Sarkar S. Charli A. Jin H. Anantharam V.. (2017). Involvement of c-Abl Kinase in microglial activation of NLRP3 inflammasome and impairment in autolysosomal system. J. Neuroimmune Pharmacol. [Epub ahead of print]. 10.1007/s11481-017-9746-5 28466394 PMC5668207

[B109] Lee M. (2013). Neurotransmitters and microglial-mediated neuroinflammation. Curr. Protein Pept. Sci. 14, 21–32. 10.2174/1389203711314010005 23441898

[B110] Lees A. J. Hardy J. Revesz T. (2009). Parkinson's disease. Lancet 373, 2055–2066. 10.1016/S0140-6736(09)60492-X 19524782

[B111] Lesage S. Brice A. (2009). Parkinson's disease: from monogenic forms to genetic susceptibility factors. Human Mol. Genet. 18, R48–R59. 10.1093/hmg/ddp012 19297401

[B112] Lesage S. Anheim M. Condroyer C. Pollak P. Durif F. Dupuits C.. (2011). Large-scale screening of the Gaucher's disease-related glucocerebrosidase gene in Europeans with Parkinson's disease. Human Mol. Genet. 20, 202–210. 10.1093/hmg/ddq454 20947659

[B113] Li G. Yang H. Zhu D. Huang H. Liu G. Lun P. (2014). Targeted suppression of chaperone-mediated autophagy by miR-320a promotes α-synuclein aggregation. Int. J. Mol. Sci. 15, 15845–15857. 10.3390/ijms150915845 25207598 PMC4200851

[B114] Li J. Xie M. Wang X. Ouyang X. Wan Y. Dong G.. (2015). Sex hormones regulate cerebral drug metabolism via brain miRNAs: Down-regulation of brain CYP2D by androgens reduces the analgesic effects of tramadol. Br. J. Pharmacol. 172, 4639–4654. 10.1111/bph.13206 26031356 PMC4594269

[B115] Li J.-Q. Tan L. Yu J.-T. (2014). The role of the LRRK2 gene in Parkinsonism. Mol. Neurodegeneration 9:47. 10.1186/1750-1326-9-47 25391693 PMC4246469

[B116] Li Y. Tomiyama H. Sato K. Hatano Y. Yoshino H. Atsumi M.. (2005). Clinicogenetic study of PINK1 mutations in autosomal recessive early-onset parkinsonism. Neurology 64, 1955–1957. 10.1212/01.WNL.0000164009.36740.4E 15955953

[B117] Liao X.-Y. Wang W.-W. Yang Z.-H. Wang J. Lin H. Wang Q.-S.. (2013). Microarray analysis of transcriptome of medulla identifies potential biomarkers for Parkinson's disease. Int. J. Genomics 2013, 7. 10.1155/2013/606919 24350239 PMC3853924

[B118] Lim W. Song G. (2014). Identification of novel regulatory genes in development of the Avian reproductive tracts. PLoS ONE 9:e96175. 10.1371/journal.pone.0096175 24763497 PMC3999111

[B119] Lin M. K. Farrer M. J. (2014). Genetics and genomics of Parkinson's disease. Genome Med. 6, 48–48. 10.1186/gm566 25061481 PMC4085542

[B120] Longhena F. Faustini G. Missale C. Pizzi M. Spano P. Bellucci A. (2017). The contribution of α-synuclein spreading to Parkinson's disease synaptopathy. Neural Plast. 2017:5012129. 10.1155/2017/5012129 28133550 PMC5241463

[B121] Lu S.-S. Gong F.-F. Feng F. Hu C.-Y. Qian Z.-Z. Wu Y.-L.. (2014). Association of microtubule associated protein tau/Saitohin (MAPT/STH) MAPT_238bp/STH Q7R polymorphisms and Parkinson's disease: a meta-analysis. Biochem. Biophys. Res. Commun. 453, 653–661. 10.1016/j.bbrc.2014.10.013 25305495

[B122] Lu X. C. Zheng J. Y. Tang L. J. Huang B. S. Li K. Tao Y.. (2015). MiR-133b promotes neurite outgrowth by targeting RhoA expression. Cell. Physiol. Biochem. 35, 246–258. 10.1159/000369692 25591767

[B123] Lu Y. Liu W. Tan K. Peng J. Zhu Y. Wang X. (2015). Genetic association of RIT2 rs12456492 polymorphism and Parkinson's disease susceptibility in Asian populations: a meta-analysis. Sci. Rep. 5:13805. 10.1038/srep13805 26334395 PMC4558715

[B124] Lücking C. B. Dürr A. Bonifati V. Vaughan J. De Michele G. Gasser T.. (2000). Association between early-onset Parkinson's disease and mutations in the Parkin gene. N. Engl. J. Med. 342, 1560–1567. 10.1056/NEJM200005253422103 10824074

[B125] Ma L. Wei L. Wu F. Hu Z. Liu Z. Yuan W. (2013). Advances with microRNAs in Parkinson's disease research. Drug Des. Dev. Ther. 7, 1103–1113. 10.2147/DDDT.S48500 24109179 PMC3792848

[B126] Ma W. Li Y. Wang C. Xu F. Wang M. Liu Y. (2016). Serum miR-221 serves as a biomarker for Parkinson's disease. Cell Biochem. Funct. 34, 511–515. 10.1002/cbf.3224 27748571

[B127] MacLeod D. Dowman J. Hammond R. Leete T. Inoue K. Abeliovich A. (2006). The familial parkinsonism gene LRRK2 regulates neurite process morphology. Neuron 52, 587–593. 10.1016/j.neuron.2006.10.008 17114044

[B128] Margis R. Margis R. Rieder C. R. M. (2011). Identification of blood microRNAs associated to Parkinsons disease. J. Biotechnol. 152, 96–101. 10.1016/j.jbiotec.2011.01.023 21295623

[B129] Marques T. M. Kuiperij H. B. Bruinsma I. B. van Rumund A. Aerts M. B. Esselink R. A.. (2016). MicroRNAs in cerebrospinal fluid as potential biomarkers for Parkinson's disease and multiple system atrophy. Mol. Neurobiol. [Epub ahead of print]. 10.1007/s12035-016-0253-0 27844283 PMC5684261

[B130] Martin I. Dawson V. L. Dawson T. M. (2011). Recent advances in the genetics of Parkinson's disease. Ann. Rev. Genomics Human Genet. 12, 301–325. 10.1146/annurev-genom-082410-101440 21639795 PMC4120236

[B131] Martin L. J. (2010). Mitochondrial and cell death mechanisms in neurodegenerative diseases. Pharmaceuticals 3, 839–915. 10.3390/ph3040839 21258649 PMC3023298

[B132] Martin L. J. Pan Y. Price A. C. Sterling W. Copeland N. G. Jenkins N. A.. (2006). Parkinson's disease α-synuclein transgenic mice develop neuronal mitochondrial degeneration and cell death. J. Neurosci. 26, 41–50. 10.1523/JNEUROSCI.4308-05.2006 16399671 PMC6381830

[B133] Martins M. Rosa A. Guedes L. C. Fonseca B. V. Gotovac K. Violante S.. (2011). Convergence of miRNA expression profiling, α-synuclein interacton and GWAS in Parkinson's disease. PLoS ONE 6:e25443. 10.1371/journal.pone.0025443 22003392 PMC3189215

[B134] Massano J. Bhatia K. P. (2012). Clinical approach to Parkinson's disease: features, diagnosis, and principles of management. Cold Spring Harb. Perspect. Med. 2:a008870. 10.1101/cshperspect.a008870 22675666 PMC3367535

[B135] Mata I. F. Shi M. Agarwal P. Chung K. A. Edwards K. L. Factor S. A. (2010). A SNCA variant associated with Parkinson's disease and plasma α-Synuclein level. Arch. Neurol. 67, 1350–1356. 10.1001/archneurol.2010.279 21060011 PMC3010848

[B136] Matsuda N. Sato S. Shiba K. Okatsu K. Saisho K. Gautier C. A.. (2010). PINK1 stabilized by mitochondrial depolarization recruits Parkin to damaged mitochondria and activates latent Parkin for mitophagy. J. Cell Biol. 189, 211–221. 10.1083/jcb.200910140 20404107 PMC2856912

[B137] McNaught K. S. P. Belizaire R. Jenner P. Olanow C. W. Isacson O. (2002). Selective loss of 20S proteasome α-subunits in the substantia nigra pars compacta in Parkinson's disease. Neurosci. Lett. 326, 155–158. 10.1016/S0304-3940(02)00296-3 12095645

[B138] Mellios N. Huang H.-S. Grigorenko A. Rogaev E. Akbarian S. (2008). A set of differentially expressed miRNAs, including miR-30a-5p, act as post-transcriptional inhibitors of BDNF in prefrontal cortex. Human Mol. Genet. 17, 3030–3042. 10.1093/hmg/ddn201 18632683 PMC2722882

[B139] Meng X.-F. Wang X.-L. Tian X.-J. Yang Z.-H. Chu G.-P. Zhang J.. (2014). Nod-Like Receptor Protein 1 Inflammasome Mediates Neuron Injury under High Glucose. Mol. Neurobiol. 49, 673–684. 10.1007/s12035-013-8551-2 24014157

[B140] Meza-Sosa K. F. Pedraza-Alva G. Pérez-Martínez L. (2014). MicroRNAs: Key triggers of neuronal cell fate. Front. Cell. Neurosci. 8:175. 10.3389/fncel.2014.00175 25009466 PMC4070303

[B141] Miñones-Moyano E. Porta S. Escaramís G. Rabionet R. Iraola S. Kagerbauer B.. (2011). MicroRNA profiling of Parkinson's disease brains identifies early downregulation of miR-34b/c which modulate mitochondrial function. Human Mol. Genet. 20, 3067–3078. 10.1093/hmg/ddr210 21558425

[B142] Miska E. A. Alvarez-Saavedra E. Townsend M. Yoshii A. Šestan N. Rakic P.. (2004). Microarray analysis of microRNA expression in the developing mammalian brain. Genome Biol. 5, R68–R68. 10.1186/gb-2004-5-9-r68 15345052 PMC522875

[B143] Moscovitz O. Ben-Nissan G. Fainer I. Pollack D. Mizrachi L. Sharon M. (2015). The Parkinson's-associated protein DJ-1 regulates the 20S proteasome. Nat. Commun. 6:6609. 10.1038/ncomms7609 25833141

[B144] Muiños-Gimeno M. Espinosa-Parrilla Y. Guidi M. Kagerbauer B. Sipil,ä T. Maron E.. (2011). Human microRNAs miR-22, miR-138-2, miR-148a, and miR-488 are associated with panic disorder and regulate several anxiety candidate genes and related pathways. Biol. Psychiatry 69, 526–533. 10.1016/j.biopsych.2010.10.010 21168126

[B145] Mushtaq G. Greig N. H. Anwar F. Zamzami M. A. Choudhry H. Shaik M. M.. (2016). Mirnas as circulating biomarkers for Alzheimer's disease and Parkinson's disease. Med. Chem. 12, 217–225. 10.2174/1573406411666151030112140 26527155 PMC6138249

[B146] Narasimhan M. Riar A. K. Rathinam M. L. Vedpathak D. Henderson G. Mahimainathan L. (2014). Hydrogen Peroxide responsive miR153 targets Nrf2/ARE cytoprotection in paraquat induced dopaminergic neurotoxicity(). Toxicol. Lett. 228, 179–191. 10.1016/j.toxlet.2014.05.020 24866057 PMC4122323

[B147] Narendra D. P. Jin S. M. Tanaka A. Suen D.-F. Gautier C. A. Shen J.. (2010). PINK1 is selectively stabilized on impaired mitochondria to activate parkin. PLoS Biol. 8:e1000298. 10.1371/journal.pbio.1000298 20126261 PMC2811155

[B148] Nicoletti G. Annesi G. Tomaino C. Spadafora P. Pasqua A. A. Annesi F.. (2002). No evidence of association between the alpha-2 macroglobulin gene and Parkinson's disease in a case–control sample. Neurosci. Lett. 328, 65–67. 10.1016/S0304-3940(02)00003-4 12123860

[B149] Nishi H. Ono K. Iwanaga Y. Horie T. Nagao K. Takemura G.. (2010). MicroRNA-15b modulates cellular ATP levels and degenerates mitochondria via Arl2 in neonatal rat cardiac myocytes. J. Biol. Chem. 285, 4920–4930. 10.1074/jbc.M109.082610 20007690 PMC2836096

[B150] Niu M. Xu R. Wang J. Hou B. Xie A. (2016). MiR-133b ameliorates axon degeneration induced by MPP+ via targeting RhoA. Neuroscience 325, 39–49. 10.1016/j.neuroscience.2016.03.042 27012608

[B151] Nuytemans K. Theuns J. Cruts M. Van Broeckhoven C. (2010). Genetic etiology of Parkinson disease associated with mutations in the SNCA, PARK2, PINK1, PARK7, and LRRK2 genes: a mutation update. Human Mutat. 31, 763–780. 10.1002/humu.21277 20506312 PMC3056147

[B152] Oertel W. Schulz J. B. (2016). Current and experimental treatments of Parkinson disease: a guide for neuroscientists. J. Neurochem. 139, 325–337. 10.1111/jnc.13750 27577098

[B153] Oki M. Hori S. Asayama S. Wate R. Kaneko S. Kusaka H. (2016). Early-onset Parkinson's disease associated with chromosome 22q11.2 deletion syndrome. Intern. Med. 55, 303–305. 10.2169/internalmedicine.55.5485 26831029

[B154] Ouchi Y. Yoshikawa E. Sekine Y. Futatsubashi M. Kanno T. Ogusu T.. (2005). Microglial activation and dopamine terminal loss in early Parkinson's disease. Ann. Neurol. 57, 168–175. 10.1002/ana.20338 15668962

[B155] Outeiro T. F. Klucken J. Bercury K. Tetzlaff J. Putcha P. Oliveira L. M.. (2009). Dopamine-induced conformational changes in alpha-Synuclein. PLoS ONE 4:e6906. 10.1371/journal.pone.0006906 19730729 PMC2731858

[B156] Perier C. Bové J. Vila M. Przedborski S. (2003). The rotenone model of Parkinson's disease. Trends Neurosci. 26, 345–346. 10.1016/S0166-2236(03)00144-9 12850429

[B157] Periquet M. Latouche M. Lohmann E. Rawal N. De Michele G. Ricard S.. (2003). Parkin mutations are frequent in patients with isolated early-onset parkinsonism. Brain 126(Pt 6), 1271–1278. 10.1093/brain/awg136 12764050

[B158] Prajapati P. Sripada L. Singh K. Bhatelia K. Singh R. Singh R. (2015). TNF-α regulates miRNA targeting mitochondrial complex-I and induces cell death in dopaminergic cells. Biochim. Biophys. Acta 1852, 451–461. 10.1016/j.bbadis.2014.11.019 25481834

[B159] Qiu L. Tan E. K. Zeng L. (2015). microRNAs and Neurodegenerative Diseases, in microRNA: Medical Evidence: From Molecular Biology to Clinical Practice, ed Santulli G. (Cham: Springer International Publishing), 85–105.

[B160] Qiu L. Zhang W. Tan E. K. Zeng L. (2014). Deciphering the function and regulation of microRNAs in Alzheimer's disease and Parkinson's disease. ACS Chem. Neurosci. 5, 884–894. 10.1021/cn500149w 25210999

[B161] Ramsey C. P. Glass C. A. Montgomery M. B. Lindl K. A. Ritson G. P. Chia L. A.. (2007). Expression of Nrf2 in neurodegenerative diseases. J. Neuropathol. Exp. Neurol. 66, 75–85. 10.1097/nen.0b013e31802d6da9 17204939 PMC2253896

[B162] Recasens A. Dehay B. Bov,é J. Carballo-Carbajal I. Dovero S. Pérez-Villalba A.. (2014). Lewy body extracts from Parkinson disease brains trigger α-synuclein pathology and neurodegeneration in mice and monkeys. Ann. Neurol. 75, 351–362. 10.1002/ana.24066 24243558

[B163] Recasens A. Perier C. Sue C. M. (2016). Role of microRNAs in the regulation of α-synuclein expression: A systematic review. Front. Mol. Neurosci. 9:128. 10.3389/fnmol.2016.00128 27917109 PMC5116472

[B164] Saha S. Guillily M. Ferree A. Lanceta J. Chan D. Ghosh J. (2009). LRRK2 modulates vulnerability to mitochondrial dysfunction in *C. elegans*. J. Neurosci. 29, 9210–9218. 10.1523/JNEUROSCI.2281-09.2009 19625511 PMC3127548

[B165] Satoh T. Okamoto S. I. Cui J. Watanabe Y. Furuta K. Suzuki M.. (2006). Activation of the Keap1/Nrf2 pathway for neuroprotection by electrophillic phase II inducers. Proc. Natl. Acad. Sci. U.S.A. 103, 768–773. 10.1073/pnas.0505723102 16407140 PMC1334635

[B166] Scarpulla R. C. (2008). Nuclear control of respiratory chain expression by nuclear respiratory factors and PGC-1-related coactivator. Ann. N. Y. Acad. Sci. 1147, 321–334. 10.1196/annals.1427.006 19076454 PMC2853241

[B167] Schapira A. H. V. Cooper J. M. Dexter D. Jenner P. Clark J. B. Marsden C. D. (1989). Mitochondrial complex i deficiency in Parkinson's disease. Lancet 333, 1269 10.1016/S0140-6736(89)92366-0 2566813

[B168] Schneider S. A. Obeso J. A. (2015). Clinical and Pathological Features of Parkinson's Disease, in Behavioral Neurobiology of Huntington's Disease and Parkinson's Disease, eds Nguyen H. H. P. Cenci M. A. (Berlin, Heidelberg: Springer Berlin Heidelberg), 205–220. 10.1007/7854_2014_317 24850081

[B169] Schrag A. Schott J. M. (2006). Epidemiological, clinical, and genetic characteristics of early-onset parkinsonism. Lancet Neurol. 5, 355–363. 10.1016/S1474-4422(06)70411-2 16545752

[B170] Schrag A. Ben-Shlomo Y. Brown R. David Marsden C. Quinn N. (1998). Young-onset Parkinson's disease revisited—clinical features, natural history, and mortality. Mov. Disord. 13, 885–894. 10.1002/mds.870130605 9827611

[B171] Schulte C. Gasser T. (2011). Genetic basis of Parkinson's disease: Inheritance, penetrance, and expression. Appl. Clin. Genet. 4, 67–80. 10.2147/TACG.S11639 23776368 PMC3681179

[B172] Sellbach A. N. Boyle R. S. Silburn P. A. Mellick G. D. (2006). Parkinson's disease and family history. Parkinsonism Relat. Disord. 12, 399–409. 10.1016/j.parkreldis.2006.03.002 16797215

[B173] Serafin A. Foco L. Blankenburg H. Picard A. Zanigni S. Zanon A.. (2014). Identification of a set of endogenous reference genes for miRNA expression studies in Parkinson's disease blood samples. BMC Res. Notes 7:715. 10.1186/1756-0500-7-715 25304816 PMC4209045

[B174] Serafin A. Foco L. Zanigni S. Blankenburg H. Picard A. Zanon A.. (2015). Overexpression of blood microRNAs 103a, 30b, and 29a in L-dopa-treated patients with PD. Neurology 84, 645–653. 10.1212/WNL.0000000000001258 25596505

[B175] Sheinerman K. S. Umansky S. R. (2013). Circulating cell-free microRNA as biomarkers for screening, diagnosis and monitoring of neurodegenerative diseases and other neurologic pathologies. Front. Cell. Neurosci. 7:150. 10.3389/fncel.2013.00150 24058335 PMC3767917

[B176] Shendelman S. Jonason A. Martinat C. Leete T. Abeliovich A. (2004). DJ-1 Is a Redox-Dependent Molecular Chaperone That Inhibits α-Synuclein Aggregate Formation. PLoS Biol. 2:e362. 10.1371/journal.pbio.0020362 15502874 PMC521177

[B177] Shi L. Wu L. Chen Z. Yang J. Chen X. Yu F.. (2015). MiR-141 Activates Nrf2-dependent antioxidant pathway via down-regulating the expression of Keap1 conferring the resistance of hepatocellular carcinoma cells to 5-fluorouracil. Cell. Physiol. Biochem. 35, 2333–2348. 10.1159/000374036 25896253

[B178] Shin J.-H. Ko H. S. Kang H. Lee Y. Lee Y.-I. Pletinkova O.. (2011). PARIS (ZNF746) repression of PGC-1α contributes to neurodegeneration in Parkinson's disease. Cell 144, 689–702. 10.1016/j.cell.2011.02.010 21376232 PMC3063894

[B179] Siebert M. Westbroek W. Chen Y.-C. Moaven N. Li Y. Velayati A.. (2014). Identification of miRNAs that modulate glucocerebrosidase activity in Gaucher disease cells. RNA Biol. 11, 1291–1300. 10.1080/15476286.2014.996085 25584808 PMC4615671

[B180] Simón-Sánchez J. Schulte C. Bras J. M. Sharma M. Gibbs J. R. Berg D.. (2009). Genome-wide association study reveals genetic risk underlying Parkinson's disease. Nat. Genet. 41, 1308–1312. 10.1038/ng.487 19915575 PMC2787725

[B181] Singleton A. B. Farrer M. Johnson J. Singleton A. Hague S. Kachergus J.. (2003). α-Synuclein locus triplication causes Parkinson's disease. Science 302, 841–841. 10.1126/science.1090278 14593171

[B182] Soreq L. Salomonis N. Bronstein M. Greenberg D. S. Israel Z. Bergman H.. (2013). Small RNA sequencing-microarray analyses in Parkinson leukocytes reveal deep brain stimulation-induced splicing changes that classify brain region transcriptomes. Front. Mol. Neurosci. 6:10. 10.3389/fnmol.2013.00010 23717260 PMC3652308

[B183] Spano M. Signorelli M. Vitaliani R. Aguglia E. Giometto B. (2015). The possible involvement of mitochondrial dysfunctions in Lewy body dementia: a systematic review. Funct. Neurol. 30, 151–158. 10.11138/FNeur/2015.30.3.151 26346695 PMC4610749

[B184] Spillantini M. G. Schmidt M. L. Lee V. M. Y. Trojanowski J. Q. Jakes R. Goedert M. (1997). α-Synuclein in Lewy bodies. Nature 388, 839–840. 9278044 10.1038/42166

[B185] Stefanis L. (2012). α-Synuclein in Parkinson's disease. Cold Spring Harb. Perspect. Med. 2:a009399. 10.1101/cshperspect.a009399 22355802 PMC3281589

[B186] Su C. Yang X. Lou J. (2016). Geniposide reduces α-synuclein by blocking microRNA-21/lysosome-associated membrane protein 2A interaction in Parkinson disease models. Brain Res. 1644, 98–106. 10.1016/j.brainres.2016.05.011 27173998

[B187] Taira T. Saito Y. Niki T. Iguchi-Ariga S. M. Takahashi K. Ariga H. (2004). DJ-1 has a role in antioxidative stress to prevent cell death. EMBO Rep. 5, 213–218. 10.1038/sj.embor.7400074 14749723 PMC1298985

[B188] Tan E.-K. Yew K. Chua E. Puvan K. Shen H. Lee E.. (2006). PINK1 mutations in sporadic early-onset Parkinson's disease. Mov. Disord. 21, 789–793. 10.1002/mds.20810 16482571

[B189] Tan M.-S. Jiang T. Tan L. Yu J.-T. (2014). Genome-wide association studies in neurology. Ann. Transl. Med. 2:124. 10.3978/j.issn.2305-5839.2014.11.12 25568877 PMC4260053

[B190] Tang G. Zhang M. Xie H. Jiang S. Wang Z. Xu L. (2002). Alpha-2 macroglobulin I1000V polymorphism in Chinese sporadic Alzheimer's disease and Parkinson's disease. Neurosci. Lett. 328, 195–197. 10.1016/S0304-3940(02)00430-5 12133586

[B191] Tatura R. Kraus T. Giese A. Arzberger T. Buchholz M. Höglinger G.. (2016). Parkinson's disease: SNCA-, PARK2-, and LRRK2- targeting microRNAs elevated in cingulate gyrus. Parkinsonism Relat. Disord. 33, 115–121. 10.1016/j.parkreldis.2016.09.028 27717584

[B192] Taylor J. M. Main B. S. Crack P. J. (2013). Neuroinflammation and oxidative stress: Co-conspirators in the pathology of Parkinson's disease. Neurochem. Int. 62, 803–819. 10.1016/j.neuint.2012.12.016 23291248

[B193] Thomas B. Mandir A. S. West N. Liu Y. Andrabi S. A. Stirling W.. (2011). Resistance to MPTP-neurotoxicity in α-Synuclein knockout mice is complemented by human α-Synuclein and associated with increased β-Synuclein and Akt activation. PLoS ONE 6:e16706. 10.1371/journal.pone.0016706 21304957 PMC3031616

[B194] Thome A. D. Harms A. S. Volpicelli-Daley L. A. Standaert D. G. (2016). microRNA-155 regulates alpha-synuclein-induced inflammatory responses in models of Parkinson disease. J. Neurosci. 36, 2383–2390. 10.1523/JNEUROSCI.3900-15.2016 26911687 PMC4764660

[B195] Tiwari P. C. Pal R. (2017). The potential role of neuroinflammation and transcription factors in Parkinson disease. Dialogues Clin. Neurosci. 19, 71–80. 28566949 10.31887/DCNS.2017.19.1/rpal PMC5442366

[B196] Todorovic M. Newman J. R. Shan J. Bentley S. Wood S. A. Silburn P. A.. (2015). Comprehensive assessment of genetic sequence variants in the antioxidant 'master regulator' Nrf2 in idiopathic Parkinson's disease. PLoS ONE 10:e0128030. 10.1371/journal.pone.0128030 26010367 PMC4444110

[B197] Valente E. M. Abou-Sleiman P. M. Caputo V. Muqit M. M. Harvey K. Gispert S.. (2004). Hereditary early-onset parkinson's disease caused by mutations in PINK1. Science 304, 1158–1160. 10.1126/science.1096284 15087508

[B198] Vallelunga A. Ragusa M. Di Mauro S. Iannitti T. Pilleri M. Biundo R.. (2014). Identification of circulating microRNAs for the differential diagnosis of Parkinson's disease and multiple system atrophy. Front. Cell. Neurosci. 8:156. 10.3389/fncel.2014.00156 24959119 PMC4051126

[B199] Varendi K. Kumar A. Härma M.-A. Andressoo J.-O. (2014). miR-1, miR-10b, miR-155, and miR-191 are novel regulators of BDNF. Cell. Mol. Life Sci. 71, 4443–4456. 10.1007/s00018-014-1628-x 24804980 PMC4207943

[B200] Vives-Bauza C. Zhou C. Huang Y. Cui M. de Vries R. L. Kim J.. (2010). PINK1-dependent recruitment of Parkin to mitochondria in mitophagy. Proc. Natl. Acad. Sci. U.S.A. 107, 378–383. 10.1073/pnas.0911187107 19966284 PMC2806779

[B201] Vogiatzi T. Xilouri M. Vekrellis K. Stefanis L. (2008). Wild type α-synuclein is degraded by chaperone-mediated autophagy and macroautophagy in neuronal cells. J. Biol. Chem. 283, 23542–23556. 10.1074/jbc.M801992200 18566453 PMC2527094

[B202] Wakabayashi K. Tanji K. Mori F. Takahashi H. (2007). The Lewy body in Parkinson's disease: molecules implicated in the formation and degradation of α-synuclein aggregates. Neuropathology 27, 494–506. 10.1111/j.1440-1789.2007.00803.x 18018486

[B203] Wake C. Labadorf A. Dumitriu A. Hoss A. G. Bregu J. Albrecht K. H.. (2016). Novel microRNA discovery using small RNA sequencing in post-mortem human brain. BMC Genomics 17:776. 10.1186/s12864-016-3114-3 27716130 PMC5050850

[B204] Wang G. van der Walt J. M. Mayhew G. Li Y.-J. Züchner S. Scott W. K.. (2008). Variation in the miRNA-433 binding site of FGF20 confers risk for Parkinson disease by overexpression of α-synuclein. Am. J. Human Genet. 82, 283–289. 10.1016/j.ajhg.2007.09.021 18252210 PMC2427225

[B205] Wang L.-L. Huang Y.-H. Yan C.-Y. Wei X.-D. Hou J.-Q. Pu J.-X.. (2016). N-acetylcysteine Ameliorates Prostatitis via miR-141 regulating Keap1/Nrf2 signaling. Inflammation 39, 938–947. 10.1007/s10753-016-0327-1 26941030

[B206] Wang T. Wang B. (2014). Association between Glutathione S-transferase M1/Glutathione S-transferase T1 polymorphisms and Parkinson's disease: a meta-analysis. J. Neurol. Sci. 338, 65–70. 10.1016/j.jns.2013.12.018 24382428

[B207] Wang X. Tan L. Lu Y. Peng J. Zhu Y. Zhang Y.. (2015). MicroRNA-138 promotes tau phosphorylation by targeting retinoic acid receptor alpha. FEBS Lett. 589, 726–729. 10.1016/j.febslet.2015.02.001 25680531

[B208] Wang X. Yan M. H. Fujioka H. Liu J. Wilson-Delfosse A. Chen S. G.. (2012). LRRK2 regulates mitochondrial dynamics and function through direct interaction with DLP1. Human Mol. Genet. 21, 1931–1944. 10.1093/hmg/dds003 22228096 PMC3315202

[B209] Wang Z. Liu J. Chen S. Wang Y. Cao L. Zhang Y.. (2011). DJ-1 modulates the expression of Cu/Zn-superoxide dismutase-1 through the Erk1/2–Elk1 pathway in neuroprotection. Ann. Neurol. 70, 591–599. 10.1002/ana.22514 21796667

[B210] Wang Z.-H. Zhang J.-L. Duan Y.-L. Zhang Q.-S. Li G.-F. Zheng D.-L. (2015). MicroRNA-214 participates in the neuroprotective effect of Resveratrol via inhibiting α-synuclein expression in MPTP-induced Parkinson's disease mouse. Biomed. Pharmacother. 74, 252–256. 10.1016/j.biopha.2015.08.025 26349993

[B211] Winfield S. L. Tayebi N. Martin B. M. Ginns E. I. Sidransky E. (1997). Identification of three additional genes contiguous to the glucocerebrosidase locus on chromosome 1q21: implications for Gaucher disease. Genome Res. 7, 1020–1026. 10.1101/gr.7.10.1020 9331372 PMC310674

[B212] Wu H. Huang M. Lu M. Zhu W. Shu Y. Cao P.. (2013). Regulation of microtubule-associated protein tau (MAPT) by miR-34c-5p determines the chemosensitivity of gastric cancer to paclitaxel. Cancer Chemother. Pharmacol. 71, 1159–1171. 10.1007/s00280-013-2108-y 23423488

[B213] Wu Y.-L. Ding X.-X. Sun Y.-H. Yang H.-Y. Sun L. (2013). Methylenetetrahydrofolate reductase (MTHFR) C677T/A1298C polymorphisms and susceptibility to Parkinson's disease: a meta-analysis. J. Neurol. Sci. 335, 14–21. 10.1016/j.jns.2013.09.006 24064257

[B214] Wu Z. Puigserver P. Andersson U. Zhang C. Adelmant G. Mootha V.. (1999). Mechanisms controlling mitochondrial biogenesis and respiration through the thermogenic coactivator PGC-1. Cell 98, 115–124. 10.1016/S0092-8674(00)80611-X 10412986

[B215] Xia J. Xu H. Jiang H. Xie J. (2015). The association between the C282Y and H63D polymorphisms of HFE gene and the risk of Parkinson's disease: a meta-analysis. Neurosci. Lett. 595(Suppl. C), 99–103. 10.1016/j.neulet.2015.04.010 25863172

[B216] Xie Y. Chen Y. (2016). microRNAs: emerging targets regulating oxidative stress in the models of Parkinson's disease. Front. Neurosci. 10:298. 10.3389/fnins.2016.00298 27445669 PMC4923223

[B217] Xiong R. Wang Z. Zhao Z. Li H. Chen W. Zhang B.. (2014). MicroRNA-494 reduces DJ-1 expression and exacerbates neurodegeneration. Neurobiol. Aging 35, 705–714. 10.1016/j.neurobiolaging.2013.09.027 24269020

[B218] Yang M. Yao Y. Eades G. Zhang Y. Zhou Q. (2011). MiR-28 regulates Nrf2 expression through a Keap1-independent mechanism. Breast Cancer Res. Treat. 129, 983–991. 10.1007/s10549-011-1604-1 21638050 PMC3752913

[B219] Yang W. Shen Y. Wei J. Liu F. (2015). MicroRNA-153/Nrf-2/GPx1 pathway regulates radiosensitivity and stemness of glioma stem cells via reactive oxygen species. Oncotarget 6, 22006–22027. 10.18632/oncotarget.4292 26124081 PMC4673142

[B220] Yang X. Liu C. Zhang J. Han H. Wang X. Liu Z.. (2015). Association of Histamine N-Methyltransferase Thr105Ile Polymorphism with Parkinson's Disease and Schizophrenia in Han Chinese: A Case-Control Study. PLoS ONE 10:e0119692. 10.1371/journal.pone.0119692 25768024 PMC4359088

[B221] Yang Z. Zhong L. Xian R. Yuan B. (2015). MicroRNA-223 regulates inflammation and brain injury via feedback to NLRP3 inflammasome after intracerebral hemorrhage. Molecular Immunology 65, 267–276. 10.1016/j.molimm.2014.12.018 25710917

[B222] Yilmaz S. G. Geyik S. Neyal A. M. Soko N. D. Bozkurt H. Dandara C. (2016). Hypothesis: do miRNAs targeting the leucine-rich repeat kinase 2 gene (LRRK2) influence parkinson's disease susceptibility? OMICS 20, 224–228. 10.1089/omi.2016.0040 27093107

[B223] Yu R.-L. Guo J.-F. Wang Y.-Q. Liu Z.-H. Sun Z.-F. Su L.. (2015). The single nucleotide polymorphism Rs12817488 is associated with Parkinson's disease in the Chinese population. J. Clin. Neurosci. 22, 1002–1004. 10.1016/j.jocn.2014.11.024 25818163

[B224] Yu X. Wang F. Zhang J. P. (2014). Meta analysis of the association of rs7702187 SNP in SEMA5A gene with risk of Parkinson's disease. Eur. Rev. Med. Pharmacol. Sci. 18, 900–904. 24706317

[B225] Zhai D. Li S. Zhao Y. Lin Z. (2014). SLC6A3 is a risk factor for Parkinson's disease: a meta-analysis of sixteen years' studies. Neurosci. Lett. 564, 99–104. 10.1016/j.neulet.2013.10.060 24211691 PMC5352947

[B226] Zhang M. An C. Gao Y. Leak R. K. Chen J. Zhang F. (2013). Emerging roles of Nrf2 and phase II antioxidant enzymes in neuroprotection. Prog. Neurobiol. 100, 30–47. 10.1016/j.pneurobio.2012.09.003 23025925 PMC3623606

[B227] Zhang Y. Cheng C. He D. Shi W. Fu C. Wang X.. (2016). Transcriptional gene silencing of dopamine D3 receptor caused by let-7d mimics in immortalized renal proximal tubule cells of rats. Gene 580, 89–95. 10.1016/j.gene.2015.12.071 26802971

[B228] Zhang Z. Cheng Y. (2014). miR-16-1 promotes the aberrant α-synuclein accumulation in Parkinson disease via targeting heat shock protein 70. Scientific World J. 2014:8. 10.1155/2014/938348 25054189 PMC4094852

[B229] Zhao N. Jin L. Fei G. Zheng Z. Zhong C. (2014). Serum microRNA-133b is associated with low ceruloplasmin levels in Parkinson's disease. Parkinsonism Relat. Disord. 20, 1177–1180. 10.1016/j.parkreldis.2014.08.016 25218846

[B230] Zheng B. Liao Z. Locascio J. J. Lesniak K. A. Roderick S. S. Watt M. L.. (2010). PGC-1α, A potential therapeutic target for early intervention in parkinson's disease. Sci. Transl. Med. 2, 52ra73-52ra73. 10.1126/scitranslmed.3001059 20926834 PMC3129986

[B231] Zhou Y. Lu M. Du R.-H. Qiao C. Jiang C.-Y. Zhang K.-Z.. (2016). MicroRNA-7 targets Nod-like receptor protein 3 inflammasome to modulate neuroinflammation in the pathogenesis of Parkinson's disease. Mol. Neurodegeneration 11, 28. 10.1186/s13024-016-0094-3 27084336 PMC4833896

