# Supplementary material for: MicroRNAs and Target Genes As Biomarkers for the Diagnosis of Early Onset of Parkinson Disease
Source: Front Mol Neurosci. 2017 Oct 31;10:352. doi: 10.3389/fnmol.2017.00352 (PMC5671573; doi:10.3389/fnmol.2017.00352)
Supplement: Supplementary file 1 [file DataSheet1.docx]

**Appendix: Gene full names for table and figure**

A2M - alpha-2 macroglobulin

ATP13A2 - ATPase 13A2

BDNF - neurotrophic factors like brain-derived neurotrophic factor

CYP2D6 - cytochrome P450 family 2 subfamily D member 6

DRD3 - dopamine receptor D3

FGF20 - fibroblast growth factor 20

GBA - glucosylceramidase beta

HSPA8 - heat shock protein family A member 8

KEAP1 - Kelch-like ECH-associated protein 1

LAMP2A - lysosomal-associated membrane protein 2A

LRRK2 - leucine rich repeat kinase 2

MAPT - microtubule-associated protein tau

NFE2L2 - nuclear factor erythroid 2-related factor 2

NFκB - nuclear factor kappa-light-chain-enhancer of activated B cells

NLRP1 – Nod-like-receptor family, pyrin domain-containing 1

NLRP3 - Nod-like-receptor family, pyrin domain-containing 3

NLRP5 - Nod-like-receptor family, pyrin domain-containing 5

PARK7 - parkinsonism associated deglycase

PGC1α - peroxisome proliferator-activated receptor gamma coactivator 1-alpha

PINK1 - PTEN induced putative kinase 1

PRKN - parkin RBR E3 ubiquitin protein ligase

RELA - NF-kB subunit/p65 protein

RHOA - RAS homolog family member A

SNCA - α-synuclein

TNFα - tumor necrosis factor alpha

UPS - ubiquitin-proteasome system

VDAC1 - voltage-dependent anion channel 1

ZNF746 - zinc finger protein 746

HNMT - histamine N-methyltransferase
